# Automated Multi-Modal MRI Segmentation of Stroke Lesions and Corticospinal Tract Integrity for Functional Outcome Prediction

**DOI:** 10.3390/tomography12030029

**Published:** 2026-02-24

**Authors:** Daniyal Iqbal, Domenec Puig, Muhammad Mursil, Hatem A. Rashwan

**Affiliations:** Department of Computer Engineering and Mathematics, Universitat Rovira i Virgili, 43007 Tarragona, Spain; domenec.puig@urv.cat (D.P.); muhammad.mursil@urv.cat (M.M.); hatem.abdellatif@urv.cat (H.A.R.)

**Keywords:** multimodal MRI, segmentation, ischemic stroke, U-Net, mRS prediction, functional outcome, deep learning, 3D lesion segmentation, TractSeg, radiomics, single-shell DWI, feature extraction

## Abstract

**Background/Objectives:** Stroke is a leading cause of long-term disability, and predicting functional outcome at discharge, such as the modified Rankin Scale (mRS), is important for guiding treatment and rehabilitation. Many existing approaches depend on advanced imaging or complex corticospinal tract (CST) segmentation from multi-shell diffusion MRI, limiting clinical feasibility. Automated lesion segmentation is also challenging due to lesion heterogeneity and MRI variability. This study proposes a clinically feasible multimodal MRI pipeline based on routine imaging. **Methods:** Lesion segmentation models were trained and evaluated on the ISLES 2022 dataset (250 training, 150 test cases). Zero-shot external evaluation was performed on 149 cases from ISLES 2024 using standard MRI sequences only. An ensemble of deep learning models (SEALS, NVAUTO, FACTORIZER) was evaluated on ISLES 2022, while SEALS alone was used for external testing. CST segmentation was performed using TractSeg on single-shell diffusion-weighted imaging. Imaging biomarkers included lesion volume, shape, ADC-based texture features, CST integrity, and lesion–CST overlap. These features were used to train machine learning models for binary mRS prediction at discharge. **Results:** The ensemble achieved a Dice score of 0.82 on ISLES 2022, while zero-shot evaluation on ISLES 2024 achieved 0.57. In exploratory analysis, CatBoost achieved the highest point estimates (accuracy 0.88, F1-score 0.87, ROC-AUC 0.83). Key predictors included lesion–CST overlap, lesion volume, surface area, dissimilarity, and contrast. **Conclusions:** This exploratory study demonstrates the feasibility of combining automated lesion segmentation with anatomically informed biomarkers using routine clinical MRI, supporting interpretable stroke outcome modelling and motivating future large-scale validation.

## Simple Summary

Stroke recovery varies widely between patients, making it difficult to estimate functional outcomes at hospital discharge. Reliable prediction methods are often based on advanced imaging techniques that are not routinely used in clinical care. This study presents a practical MRI-based framework that relies only on standard imaging commonly acquired in stroke patients. By automatically identifying stroke lesions and assessing their relationship to key motor pathways, we derived interpretable imaging markers linked to functional outcome. Our findings suggest that routinely available MRI data can support clinically meaningful and transparent prediction of short-term stroke outcomes, highlighting the potential for broader clinical adoption.

## 1. Introduction

Ischemic stroke remains one of the leading causes of disability and mortality worldwide, and inadequate prognostication can hinder optimal rehabilitation and treatment planning [1]. Its impact is influenced by factors such as age, environment, socioeconomic status, and regional disparities, all of which affect patient independence and quality of life [2]. Early and accurate prediction of functional outcomes is therefore critical for guiding individualised therapy. Among standardised clinical measures, the modified Rankin Scale (mRS) is widely accepted for assessing post-stroke disability [3].

Magnetic resonance imaging (MRI) has become central to outcome prediction due to its superior sensitivity and detailed depiction of brain tissue compared to computed tomography (CT). MRI-derived biomarkers such as lesion size, location, morphology, texture, and corticospinal tract (CST) integrity offer valuable insights into the extent and functional consequences of neural damage [4,5,6]. These biomarkers enable the precise assessment of residual brain functionality and guide targeted rehabilitation strategies aimed at improving mRS outcomes at discharge.

Despite these advantages, several barriers limit the integration of advanced MRI techniques into routine clinical workflows. Multi-shell diffusion MRI protocols, for example, require long acquisition times, specialised hardware, and complex processing pipelines, making them impractical for widespread adoption [7]. Moreover, many studies focus on single imaging modalities or isolated features, missing the added prognostic value of combining multimodal data and diverse feature types [8,9,10].

To address these challenges, we developed a clinically deployable and fully automated dual-stream pipeline that operates on routinely acquired single-shell MRI sequences (DWI, ADC, and FLAIR) in acute stroke care. The framework combines advanced automated lesion segmentation with anatomically informed CST overlap analysis to extract a comprehensive set of imaging biomarkers, including volumetric, morphological, ADC-derived texture, and lesion–CST overlap features. These biomarkers capture both the extent and anatomical relevance of brain damage, enabling an interpretable and feature-based machine learning model for functional outcome prediction. Feature importance analysis is incorporated to support interpretability and to identify imaging-derived predictors that may be associated with functional outcome. By integrating segmentation, tract-specific analysis, and clinically feasible MRI acquisition, our approach aims to provide a reproducible and pragmatic framework for investigating imaging-based risk stratification in stroke management, rather than a fully validated clinical decision-support system.

The remainder of this paper is organised as follows. Section 2 demonstrates the related work. Section 3 describes the datasets, preprocessing steps, and the proposed pipeline, including lesion and CST segmentation, feature extraction, and the machine learning framework. Section 4 presents the experimental setup, performance metrics, and predictive results. Section 5 discusses the clinical implications of the findings, limitations, and directions for future research. Finally, Section 6 concludes the paper.

## 2. Related Work

Predicting functional outcomes after ischemic stroke from neuroimaging has attracted increasing attention in recent years. Advances in MRI-based lesion characterisation, white-matter tract mapping, and radiomics have enabled more precise prognostic modelling. By quantifying lesion size, morphology, and involvement of motor pathways such as the CST regions, researchers have linked imaging biomarkers to patient disability, most commonly measured by mRS. Parallel developments in deep learning have automated the extraction of these features, raising the possibility of rapid, reproducible, and clinically relevant outcome prediction. However, the literature remains fragmented; lesion segmentation, tractography, and predictive modelling are often developed in isolation, and many pipelines are not optimised for real-world deployment.

In the following subsections, we review relevant work across three domains: (1) ischemic stroke lesion segmentation and CST mapping, (2) MRI-based prediction of functional outcomes, and (3) approaches targeting real-world clinical integration. We then summarise these studies in Table 1 and highlight the technical and clinical gaps that motivate our approach.

### 2.1. Ischemic Stroke Lesion Segmentation and CST Mapping

Accurate segmentation of the lesions is essential for downstream biomarker extraction and outcome prediction. Early work by Tomita et al. (2020) [11] used a 52-layer 3D residual U-Net to segment chronic infarcts on T1-weighted MRI, achieving a mean Dice of 0.64. Subsequent U-Net variants have leveraged multimodal MRI to improve accuracy; Gheibi et al. (2023) [12] reported 0.85 Dice on an internal dataset, while the ISLES 2022 challenge [13] benchmarked models on 250 annotated cases, with top ensembles reaching 0.82 Dice. Large-scale training across sites, as in Ryu et al. (2025) [14], further improved robustness (Dice 0.70) but still struggled with small or hyperacute infarcts and brainstem lesions. Domain shifts between scanners and protocols remain a challenge.

In parallel, deep learning–based CST segmentation tools have matured. TractSeg [15] can delineate major white-matter bundles from diffusion MRI, including in pathological brains. Kim et al. (2024) [16] applied a squeeze–excitation U-Net to segment white-matter hyperintensities, achieving Dice 0.722, while Moshe et al. (2022) [17] demonstrated reliable CST delineation (Dice 0.63) with high test–retest reproducibility. Overlaying lesion masks with CST maps enables quantification of motor pathway involvement, a clinically important biomarker, but most studies quantify lesion–CST overlap only post hoc, using simple overlap measures that fail to capture topological details [18]. As a result, predictors often overemphasise lesion volume while overlooking strategically disabling tract damage.

### 2.2. MRI-Based Prediction of Functional Outcome (mRS)

Numerous studies have linked MRI-derived lesion characteristics to mRS outcomes at discharge. Radiomics-based approaches, such as Quan et al. (2021) [19], have combined ADC and FLAIR texture features with clinical variables in logistic regression models, achieving high discrimination (AUC 0.864 external). Zhang et al. (2022) [20] reported similar gains by integrating ADC-based texture with clinical data, while Wei et al. (2024) [21] showed that combining DWI and ADC radiomics improved performance over single-modality features. More recent deep learning methods, like the multi-task nnU-Net of Park et al. (2024) [22], jointly segment lesions and predict mRS at discharge, outperforming traditional scores. Texture analysis of white-matter hyperintensities has also shown predictive value, with Xia et al. (2025) [23] achieving an AUC of 0.94 using SVM-based radiomics. Despite these promising results, limitations persist; most studies have modest sample sizes, and tract-specific biomarkers such as CST overlap are rarely incorporated. Interpretability remains limited, with few works going beyond generic visualisation techniques.

### 2.3. Addressing Real-World Barriers

While accuracy is critical, clinical adoption also depends on practicality. Many high-performing pipelines require multi-shell diffusion MRI [15,24], complex preprocessing [14,22], or extended runtimes, which are incompatible with acute care constraints where rapid decision-making is essential. Moreover, models are often trained on single-centre datasets [19,20], limiting their generalisation to heterogeneous patient populations and scanner protocols. Recent work has begun addressing these issues by streamlining inputs, e.g., relying on single-shell DWI and ADC [21] and optimising computational efficiency through containerised or cloud-based frameworks [25,26]. Prior work on CST involvement is limited to tract-specific biomarkers. Most models rely on global lesion metrics, while CST involvement, when considered, is often reduced to simple overlap measures. Such coarse quantification overlooks spatial and topological nuances (e.g., sub-pathway disruptions beyond M1) that critically shape motor recovery [27]. This risks overstating lesion volume while underestimating strategically disabling tract damage. However, fully integrated, clinically deployable solutions remain rare. In particular, methods that combine robust multimodal segmentation, tract-based biomarker extraction, interpretable machine learning, and compatibility with standard imaging protocols are still lacking [16,17].

#### Handling Data Limitations and Interpretability

Recent methods have begun tackling the twin challenges of limited and imbalanced stroke datasets using generative modelling. For instance, conditional tabular GANs (ctGANs) have proven effective in rare-disease gait classification by generating synthetic, explainable samples that enhance classifier performance without compromising interpretability [28]. In ischemic stroke contexts, hybrid GAN architectures (e.g., WGAN and ACGAN) have been deployed to oversample underrepresented patient subgroups, improving training balance and sample diversity [29]. Generative models have also been used to synthesise perfusion maps from original imaging sequences, achieving expert-level fidelity (SSIM up to 0.99) and promising swift, automated feature extraction [30]. Equally critical for clinical deployment is model interpretability. A growing consensus in medical AI flags interpretability, reproducibility, shareability, and accountability as foundational design criteria favouring intrinsically interpretable models (e.g., sparse kernels or prototype-based learners) over opaque deep networks. Complementary XAI tools like SHAP and LIME further bolster understandability and trust in prognosis-oriented ML systems [31].

**Table 1:** Summary of the recent literature on automated segmentation of stroke lesions and CST integrity for outcome prediction.

Literature	Cohort/Data	Goals/Models	Results/Limitations
Tomita et al., 2020 [11]	239 chronic T1-weighted MRI stroke patients	**Goal**: Automated infarct segmentation in chronic stroke **Model**: 3D Residual U-Net	**Result**: Dice 0.64, robust on mid-sized lesions **Limitation**: Under-segments very small infarcts; single-centre retrospective; no external testing
Gheibi et al., 2023 [12]	110 acute DWI, FLAIR, T1 (private)	**Goal**: Segment acute stroke lesions for outcome modelling **Model**: 3D CNN-Res with focal + Dice loss	**Result**: Dice 0.85 **Limitation**: No external validation; small, vendor-limited dataset
ISLES 2022 Challenge [32]	250 annotated stroke MRIs (DWI/ADC/FLAIR)	**Goal**: Benchmark generalizable lesion segmentation **Model**: Ensemble of 3D U-Nets + Factorizer fusion	**Result**: Dice 0.82 **Limitation**: focus only on segmentating the lesion
Ryu et al., 2025 [14]	10,820 multisite DWI AIS cases	**Goal**: Assess segmentation across diverse acute infarcts **Model**: 3D U-Net (Dice + cross-entropy loss)	**Result**: Dice 0.70 **Limitation**: Poor on brainstem/hyperacute (<3 h) strokes; DWI only
Kim et al., 2024 [16]	8421 AIS cases (FLAIR/ADC WMH)	**Goal**: Quantify CST involvement and predict motor impairment risk **Model**: 2D U-Net (Dice + focal loss)	**Result**: Dice 0.722 **Limitation**: 2D slices lose 3D context
Moshe et al., 2022 [17]	649 DTI scans	**Goal**: Segment corticospinal tract for motor outcome assessment **Model**: TractSeg automated delineation	**Result**: Dice 0.63 **Limitation**: Does not quantify tract integrity or functional connectivity
Quan et al., 2021 [19]	190 AIS ADC + FLAIR images	**Goal**: Predict unfavourable 3-month mRS for care planning **Model**: Radiomic features + traditional classifiers	**Result**: AUC 0.864 (internal) **Limitation**: Single-centre data; “external” set same institution
Zhang et al., 2022 [20]	103 anterior circulation ADC scans	**Goal**: Stratify 90-day disability using imaging + clinical models **Model**: Random Forest and logistic regression	**Result**: AUC 0.83 **Limitation**: Small; no external validation; only anterior strokes
Wei et al., 2024 [21]	461 AIS DWI + ADC cases	**Goal**: Early prediction of poor functional outcome **Model**: Multivariate logistic regression	**Result**: AUC 0.825 **Limitation**: Variable protocols; dichotomised outcome
Park et al., 2024 [22]	5429 AIS DWI images	**Goal**: Joint lesion segmentation and 90-day mRS prediction **Model**: Multi-task 3D nnU-Net	**Result**: mRS AUC 0.81 **Limitation**: Lacks perfusion biomarkers; binary mRS loses granularity
Xia et al., 2025 [23]	202 AIS FLAIR WMH images	**Goal**: Classify poor outcome via lesion heterogeneity **Model**: SVM on radiomic texture and shape features	**Result**: AUC 0.939 **Limitation**: Single-centre cohort; no diffusion core data

## 3. Materials and Methods

### 3.1. Datasets

We used two cohorts: the public ISLES 2022 ischemic stroke dataset and the ISLES 2024 acute ischemic stroke dataset, which serve distinct purposes in this study.

The ISLES 2022 dataset comprises 400 cases, of which 250 cases were used for training and 150 cases for testing the lesion segmentation models. This dataset provides expert-annotated ischemic lesion masks but does not include functional outcome measures and was therefore used exclusively for lesion segmentation model development and evaluation.

The ISLES 2024 dataset was used for external evaluation and outcome modelling. Although the full ISLES 2024 cohort consists of 249 subjects (149 publicly released training cases and 100 hidden test cases), the official test set does not provide publicly available MRI data, ground-truth lesion masks, or modified Rankin Scale (mRS) labels. Consequently, only the 149 publicly released cases with complete imaging and outcome annotations were included in this study. These cases were treated as a fixed external cohort.

Importantly, no ISLES 2024 data were used for training or fine-tuning the lesion segmentation models. Instead, ISLES 2024 was employed to (i) perform zero-shot external evaluation of lesion segmentation and (ii) train and evaluate machine learning models for functional outcome (mRS) prediction, as functional outcome labels are not available in ISLES 2022.

The ISLES 2024 dataset includes multiple imaging modalities acquired as part of routine clinical stroke assessment, namely non-contrast CT (NCCT), CT angiography (CTA), CT perfusion (CTP; including Tmax, CBF, CBV, and MTT maps), and follow-up MRI comprising diffusion-weighted imaging (DWI) and apparent diffusion coefficient (ADC) maps. As the lesion segmentation framework was developed using MRI data, only the follow-up MRI modalities (DWI and ADC) were utilised in the present analysis. CT-based modalities were not considered further.

For functional outcome prediction at discharge, the ISLES 2024 cohort exhibited a binary mRS distribution of 54 patients with a favourable outcome (mRS ≤ 2) and 43 patients with an unfavourable outcome (mRS > 2) among cases with available outcome labels. Cases with missing mRS information were excluded from outcome prediction analyses and are reported explicitly in Section 3.4. All imaging data were fully anonymised by the dataset providers and processed in accordance with the respective dataset guidelines. A detailed comparison of imaging characteristics and acquisition heterogeneity between ISLES 2022 and ISLES 2024 is provided in Table 2.

#### 3.1.1. ISLES 2022 Dataset

The ISLES 2022 [32] is a multi-centre MRI benchmark specifically designed for ischemic stroke lesion segmentation. It comprises 400 cases acquired across three European stroke centres using scanners from different vendors (Philips and Siemens) and field strengths (1.5 T and 3 T). The dataset is split into 250 publicly available training cases and 150 hidden test cases. Each case includes co-registered and skull-stripped magnetic resonance images consisting of diffusion-weighted imaging(DWI: b = 1000 s/mm^2^), apparent diffusion coefficient (ADC) maps, and fluid-attenuated inversion recovery (FLAIR) sequences, provided in NIfTI format. Expert-annotated lesion masks are available for all training cases, together with scanner and acquisition metadata. To encourage generalisation, the dataset was constructed such that training data originate from two centres, while the test data come from a third, unseen centre. Lesions vary widely in size, number, and spatial distribution, making ISLES 2022 a challenging and widely adopted benchmark for evaluating automated stroke lesion segmentation methods. The ISLES 2022 dataset was used exclusively for training and evaluating lesion segmentation models, as it provides high-quality expert lesion annotations but does not include functional outcome measures. No outcome prediction models were trained on ISLES 2022.

#### 3.1.2. ISLES 2024 Dataset

The ISLES 2024 dataset [33] includes patients aged 18 years or older who underwent acute or sub-acute stroke imaging as part of routine clinical care, followed by intracranial interventional reperfusion therapy. The full dataset consists of 249 cases, of which 149 cases are publicly available with complete imaging and outcome annotations. Imaging protocols include non-contrast CT (NCCT), CT angiography (CTA), CT perfusion (CTP; including Tmax, cerebral blood flow (CBF), cerebral blood volume (CBV), and mean transit time (MTT) maps), and follow-up magnetic resonance imaging performed 2–9 days after stroke onset. The follow-up MRI includes, at minimum, diffusion-weighted imaging (DWI) and corresponding apparent diffusion coefficient (ADC) maps. Data were acquired across multiple comprehensive stroke centres using scanners from different vendors (Siemens and Philips) and field strengths (1.5 T and 3 T), resulting in substantial heterogeneity in acquisition parameters and preprocessing. This variability reflects real-world clinical conditions and supports the evaluation of model robustness and generalisability. In addition to imaging data, the ISLES 2024 dataset provides discharge modified Rankin Scale (mRS) scores for a subset of patients, enabling functional outcome analysis. Demographic and clinical summary statistics for this cohort are reported in Table 3. The ISLES 2024 dataset served a dual role. First, it was used for external, zero-shot evaluation of lesion segmentation, where the trained segmentation model was applied without retraining or fine-tuning to assess generalisation under realistic deployment conditions. Second, ISLES 2024 was the only dataset used for training and evaluating functional outcome prediction models, as it includes discharge mRS annotations. Imaging-derived features extracted from the segmented lesions and corticospinal tract were used to train and test machine learning models for binary mRS prediction.

### 3.2. Data Preparation

First, we collected two types of MRI images, diffusion-weighted (DWI) and apparent diffusion coefficient (ADC). Fluid-attenuated inversion recovery (FLAIR) images were available for ISLES 2022 and were used during lesion segmentation model training; however, FLAIR was not consistently available for ISLES 2024 and was therefore not required for external evaluation. Before further processing, all images underwent a manual quality control step to verify file integrity, orientation consistency, and compatibility with the processing pipeline.

Because only single-shell diffusion data were available, it was not possible to estimate subject-specific multi-compartment diffusion models. Instead, we employed a population-averaged “global fibre peaks” template to approximate voxel-wise fibre orientations for each subject. This approach provides a pragmatic solution for tract-based analysis when advanced diffusion acquisitions are unavailable and has been used in prior work for anatomically informed tract segmentation under constrained imaging conditions. Using these estimated fibre orientation peaks, we delineated the left and right corticospinal tracts (CSTs) for each subject. Tract segmentation was performed in a Windows-based environment using MSYS2 (v14.2.0), MRtrix3 (v3.0.4) for diffusion preprocessing, and TractSeg (v2.9) for automated tract delineation.

With both lesion masks and CST segmentations available, we extracted volumetric, spatial, and overlap-based features describing lesion extent and its anatomical relationship to the CST. These tract-based measures are intended as surrogate anatomical descriptors rather than precise estimates of white matter integrity, given the use of single-shell diffusion data and fully automated segmentation. All extracted imaging features were assembled into a structured tabular dataset and linked to the corresponding mRS score at discharge. This feature table formed the basis for subsequent exploratory machine learning analyses aimed at modelling functional outcome at discharge.

### 3.3. Methods

**Figure 1:**
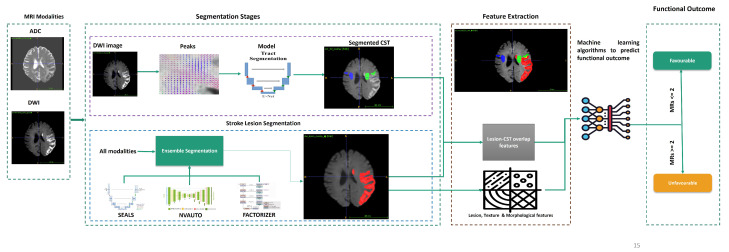
Schematic representation of the proposed outcome prediction framework. The pipeline starts with multimodal MRI inputs (DWI and ADC). Lesion segmentation is performed using an ensemble of deep learning models (SEALS, NVAUTO, FACTORIZER) when trained and evaluated on ISLES 2022. For external evaluation on ISLES 2024, segmentation is performed using the SEALS model only due to modality availability constraints. Subsequent steps include CST segmentation using TractSeg and spatial analysis of lesion–tract relationships. Quantitative imaging biomarkers, comprising lesion volume, morphological descriptors, texture features, and lesion–CST overlap metrics, are extracted and used for exploratory ML–based modelling of favourable and unfavourable functional outcomes as measured by the mRS at discharge.

Figure 1 provides an overview of the proposed fully automated and modular modelling framework. The pipeline is designed to transform routine clinical MRI into anatomically informed imaging biomarkers in a transparent and interpretable manner. It integrates multiple processing stages–lesion segmentation, CST extraction, feature computation, and machine learning–into a cohesive workflow suitable for exploratory outcome analysis.

The framework begins with multimodal MRI inputs, specifically DWI and ADC maps (512, 595, 75). On ISLES 2022, stroke lesions are segmented using an ensemble of deep learning models (SEALS, NVAUTO, FACTORIZER) [13], generating lesion masks in approximately one minute per case. For ISLES 2024, lesion segmentation is performed using the SEALS model only, reflecting a zero-shot external evaluation setting. DWI volumes (512, 595, 75) are preprocessed to compute local fibre peaks, which serve as input to a U-Net for corticospinal tract (CST) segmentation (512, 595, 75) using MRtrix3 [34], which takes approximately 0.5 min, and is segmented via TractSeg [15], which takes approximately 1 min.

From the resulting lesion and CST segmentations, we extract 19 imaging biomarkers, including lesion volume, spatial distribution, shape descriptors, radiomic texture measures, and lesion–CST overlap metrics. These features are assembled into structured tabular data and used to train machine learning models for binary mRS outcome modelling at discharge. Model inference requires less than one second per case. Each component of the pipeline is described in detail in the following subsections.

#### 3.3.1. Lesion Segmentation

Lesion segmentation was achieved through an ensemble-based segmentation strategy comprising (SEALS [35], NVAUTO [36], and FACTORIZER [37]), trained and internally evaluated on the ISLES 2022 dataset, where DWI, ADC, and FLAIR modalities were available. The three segmentation models included in the ensemble were selected to reflect diverse architectural and methodological assumptions, thereby reducing sensitivity to any single modelling bias. SEALS [35] is an atlas-guided deep learning framework that integrates spatial priors with convolutional representations, enabling robust lesion localisation across heterogeneous anatomical presentations. NVAUTO [36] employs a patch-based convolutional neural network architecture, focusing on local intensity patterns and contextual neighbourhood information to capture fine-grained lesion details. In contrast, FACTORIZER [37] adopts a probabilistic factorisation strategy, modelling lesion appearance through latent components and enabling robust segmentation under variable contrast and noise conditions. By combining these complementary approaches, the ensemble leverages global anatomical consistency (SEALS), local appearance sensitivity (NVAUTO), and probabilistic robustness (FACTORIZER), which together improve segmentation stability across diverse imaging conditions.

The outputs of the three models are integrated using voxel-wise majority voting, a conservative ensembling strategy commonly used in medical image segmentation. For each voxel, a binary lesion label is independently predicted by each model. The final lesion label is assigned only when at least two of the three models agree on a lesion classification. Voxels without majority agreement are assigned as non-lesion, thereby limiting the propagation of uncertain predictions. This strategy prioritises robustness and specificity over aggressive lesion inclusion and helps mitigate false positives arising from model-specific artefacts.

**Figure 2:**
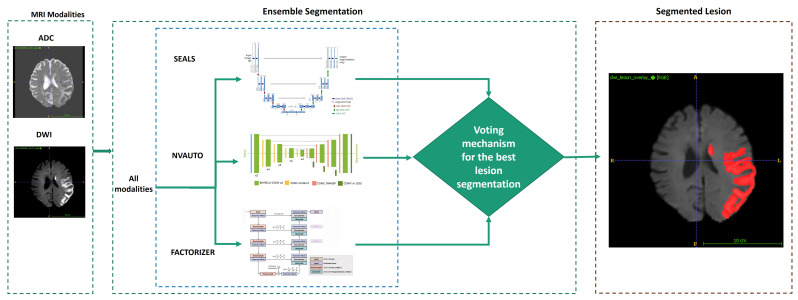
Lesion segmentation workflow for ISLES 2022 and ISLES 2024 datasets. For ISLES 2022, lesion segmentation was performed using an ensemble of SEALS, NVAUTO, and FACTORIZER on DWI, ADC, and FLAIR inputs, with voxel-wise majority voting used to derive the final lesion mask. For ISLES 2024, only DWI and ADC modalities were available; therefore, lesion segmentation was performed using the SEALS model only, reflecting a zero-shot external evaluation scenario.

For external evaluation on the ISLES 2024 dataset, modality availability differed substantially from that of ISLES 2022. In particular, FLAIR images were not consistently available across the ISLES 2024 cohort. As both NVAUTO and FACTORIZER require FLAIR input, these models could not be applied in this setting. Consequently, lesion segmentation for ISLES 2024 was performed exclusively using the SEALS model, which operates on routinely available DWI and ADC modalities. This configuration therefore represents a zero-shot external evaluation under realistic clinical constraints, where segmentation performance is assessed without retraining or ensembling. While this precludes the use of voxel-wise majority voting and is expected to result in reduced segmentation accuracy compared to the internal ISLES 2022 evaluation, it more accurately reflects real-world deployment scenarios in which modality availability is limited and heterogeneous. Figure 2 illustrates the segmentation workflow.

#### 3.3.2. Spatial and Intensity Feature Extraction

In addition to lesion volume and shape descriptors, a set of spatial and intensity-based features was extracted from the segmented lesion masks to characterise lesion location and internal signal properties.

**Lesion location (Quadrant-Based Analysis):** Lesion location was analysed by partitioning the brain volume into four anatomical quadrants: Left Anterior (LA), Left Posterior (LP), Right Anterior (RA), and Right Posterior (RP). The brain is divided along the mid-sagittal and mid-coronal planes to define these quadrants.

The mid-sagittal and mid-coronal planes are determined as follows:

(1) $$x_{\mathrm{mid}}=\frac{\text{brain\_shape[0]}}{2},$$

(2) $$z_{\mathrm{mid}}=\frac{\text{brain\_shape[2]}}{2},$$

where brain_shape[0] and brain_shape[2] denote the number of voxels along the x and z dimensions of the brain volume, respectively.

**Intensity-Based Lesion Descriptors:** In addition to spatial localisation, ADC-derived intensity features were extracted to capture intra-lesional signal characteristics. The following descriptors were computed:

**Lesion Centroid**—The intensity-weighted geometric centre of the lesion was computed as:

(3) $$\mathrm{Centroid}=\left(\frac{\sum x\cdot I(x,y,z)}{\sum I(x,y,z)},\frac{\sum y\cdot I(x,y,z)}{\sum I(x,y,z)},\frac{\sum z\cdot I(x,y,z)}{\sum I(x,y,z)}\right),$$

where I(x,y,z) denotes the voxel intensity at spatial coordinates (x,y,z).

**Maximum Intensity**—The highest signal intensity within the lesion mask:

(4) MaxIntensity=max(I(x,y,z)).

**Average Intensity**—The mean signal intensity across all voxels in the lesion:

(5) $$\mathrm{AvgIntensity}=\frac{\sum I(x,y,z)}{\sum L(x,y,z)},$$

where L(x,y,z) is the binary lesion mask, with L=1 for lesion voxels and L=0 otherwise.

#### 3.3.3. Morphological Feature Extraction

Morphological features characterise the geometric structure, spatial extent, and boundary complexity of the lesion. The following descriptors are computed from the binary lesion mask:

**Lesion Volume**—The total volume of infarcted tissue, expressed in millilitres:

(6) $$V_{\mathrm{lesion}}=\frac{\sum L\left(x,y,z\right)\cdot v_{\mathrm{voxel}}}{1000},$$

where L(x,y,z) is the binary lesion mask and $v_{\mathrm{voxel}}$ is the voxel volume in ${\mathrm{mm}}^{3}$.

**Surface Area**—Calculated via a marching cubes algorithm applied to the 3D lesion mesh:

(7) SurfaceArea=mesh_surface_area(vertices, faces).

**Sphericity**—A dimensionless measure comparing the lesion’s shape to a perfect sphere:

(8) $$\mathrm{Sphericity}=\frac{\pi^{1/3}{\left(6V_{\mathrm{lesion}}\right)}^{2/3}}{A_{\mathrm{lesion}}}$$

**Solidity**—The ratio of the lesion volume to its convex hull volume:

(9) $$\mathrm{Solidity}=\frac{V_{\mathrm{lesion}}}{V_{\mathrm{convex}\;\mathrm{hull}}}.$$

**Elongation**—A measure of anisotropy defined as the ratio of the minor axis length to the major axis length:

(10) $$\mathrm{Elongation}=\frac{\mathrm{MinorAxisLength}}{\mathrm{MajorAxisLength}}.$$

**Compactness**—A normalised metric relating surface area to volume:

(11) $$\mathrm{Compactness}=\frac{{\mathrm{SurfaceArea}}^{3}}{36\pi \cdot V_{\mathrm{lesion}}^{2}}.$$

These morphological descriptors were selected because they capture complementary and clinically interpretable aspects of lesion geometry that are clinically relevant to stroke prognosis. Lesion volume is a strong predictor of outcome, with larger infarcts generally associated with more severe deficits. Surface area and compactness quantify lesion boundary complexity, which can indicate heterogeneous tissue damage. Sphericity and elongation describe shape regularity and anisotropy, potentially reflecting the lesion’s spread along vascular territories or white matter tracts. Solidity, measuring how closely the lesion fills its convex boundary, may indicate the presence of fragmented or satellite lesions. Together, these features provide a detailed geometric fingerprint of the infarct, enriching the prediction model beyond simple volumetric measures.

#### 3.3.4. Texture Feature Extraction

To quantify the intrinsic heterogeneity of the ischemic lesion, a texture analysis was performed exclusively on ADC images within the segmented lesion volume. ADC was selected because it provides quantitative diffusion information and was consistently available across both datasets. The extracted texture features describe second-order statistical relationships between voxel intensities and are intended to capture spatial variations in lesion appearance. This process provided second-order statistical features that describe the spatial relationships between voxel intensities.

Prior to texture computation, voxel intensities within the lesion mask were independently normalised to an 8-bit integer range (0–255) to reduce the influence of absolute intensity variability and to ensure numerical stability across subjects. Texture features were extracted using a two-dimensional Grey-Level Co-Occurrence Matrix (GLCM) approach applied on a slice-wise basis. For every slice, the number of discrete grey levels was set to 256. A GLCM was computed for three pixel distances (d = 1, 2, 3) and across four directions (0°, 45°, 90°, 135°), generating a matrix that tabulates how often pairs of pixels with specific values occur at a given spatial relationship. These matrices were made symmetric and normalised to probabilities.

From each directional GLCM, six standard Haralick features were computed: Contrast, Dissimilarity, Homogeneity, Angular Second Moment (ASM), Energy, and Correlation. To ensure rotational invariance, feature values were averaged across the four directions for each slice. Shannon Entropy was computed directly from the slice-wise intensity histogram rather than from the GLCM. This process yielded a set of seven texture features per silce. To obtain patient-level descriptors, feature values were averaged across all axial slices containing lesion voxels using the arithmetic mean. The resulting per-patient texture feature vector was subsequently used for exploratory outcome modelling. From the normalised GLCM p(i,j), where p(i,j) denotes the probability of grey levels *i* and *j* occurring together, we extracted the following standard radiomic features:

(12) $$\begin{array}{cc} \mathrm{Contrast} & =\sum_{i,j}{\left(i-j\right)}^{2}\cdot p\left(i,j\right) \end{array}$$

(13) $$\begin{array}{cc} \mathrm{Dissimilarity} & =\sum_{i,j}\left|i-j\right|\cdot p\left(i,j\right) \end{array}$$

(14) $$\begin{array}{cc} \mathrm{Homogeneity} & =\sum_{i,j}\frac{1}{1+|i-j|}\cdot p\left(i,j\right) \end{array}$$

(15) $$\begin{array}{cc} \mathrm{ASM} & =\sum_{i,j}p{\left(i,j\right)}^{2} \end{array}$$

(16) $$\begin{array}{cc} \mathrm{Energy} & =\sqrt{\mathrm{ASM}} \end{array}$$

(17) $$\begin{array}{cc} \mathrm{Correlation} & =\frac{\sum_{i,j}\left(i-\mu_{i}\right)\left(j-\mu_{j}\right)\cdot p\left(i,j\right)}{\sigma_{i}\sigma_{j}} \end{array}$$

(18) $$\begin{array}{cc} \mathrm{Entropy} & =-\sum_{i,j}p\left(i,j\right)\cdot \log_{2}p\left(i,j\right) \end{array}$$

where *i* and *j* are the grey-level indices of the matrix, $\mu_{i}$ and $\mu_{j}$ are the means of the marginal distributions of *i* and *j*, and $\sigma_{i}$ and $\sigma_{j}$ are the corresponding standard deviations. Each feature captures different aspects of lesion texture: *Contrast* and *Dissimilarity* reflect local intensity variation, *Homogeneity* and *ASM* measure uniformity, *Energy* summarises pattern regularity, *Correlation* describes the degree of linear dependency between voxel intensities, and *Entropy* quantifies signal randomness. Together, these descriptors provide a compact statistical characterisation of ADC signal heterogeneity within the lesion and are used as imaging-derived features in the subsequent exploratory outcome modelling.

#### 3.3.5. Fibre Peak Estimation Using MRtrix3

To perform tractography (CST), we first estimated voxel-wise fibre orientation distributions (FODs) from diffusion-weighted imaging (DWI) data. This was achieved using MRtrix3 [34], a widely used software for diffusion MRI processing and tractography. Because only single-shell DWI acquisitions were available, we employed constrained spherical deconvolution (CSD) with regularisation to resolve crossing fibres and infer fibre orientations.

The DWI volumes were first converted to MRtrix image format (MIF), incorporating associated b-values and b-vectors for gradient direction mapping. A population-averaged ‘global peaks’ template was used to initialise peak directions for FOD estimation, allowing for anatomically consistent peak reconstruction across subjects.

After generating the FODs, the principal fibre directions, also known as ‘peaks’, were extracted from each voxel. These peaks provide critical orientation information needed for downstream tract segmentation. Affine registration was applied to align the generated peaks with each subject’s DWI space, ensuring anatomical accuracy.

**Figure 3:**
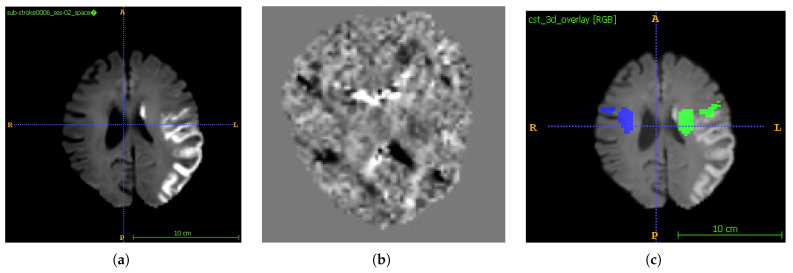
(**a**) Original DWI image from which the peaks are generated (**b**) Peaks image. Fibre orientation distributions (FODs) computed from the ISLES 2024 dataset diffusion-weighted imaging (DWI) using MRtrix3’s constrained spherical deconvolution (CSD) algorithm. Each line indicates the primary diffusion directions within a voxel, enabling the modelling of complex fibre configurations such as crossing or fanning fibres. (**c**) represents CST segmentation results obtained using TractSeg, which are shown for both hemispheres. The tractography was performed on preprocessed ISLES 2024 dataset diffusion-weighted images (DWIs). TractSeg was used to generate white matter bundle segmentations based on deep learning from the FOD peaks. It represents the left CST and right CST on the mid-slice of DWI in axial view. This visualisation serves as an anatomical reference for assessing white matter architecture and for further quantification of stroke impact on motor pathways.

Figure 3 visualises the resulting FOD peaks, where each line indicates a dominant fibre direction in a voxel. These peaks form the input to the CST segmentation pipeline using TractSeg (see Section 3.3.6).

#### 3.3.6. Corticospinal Tract (CST) Segmentation

CST segmentation was performed using TractSeg [15], a deep learning-based framework that accurately delineates major white matter tracts from FODs. As input, we used the subject-specific peak images generated during the FOD estimation step (see Section 3.3.5). The commands for generating the CST are mentioned in Table 4.

TractSeg processes these peak maps to produce binary masks of the left and right CSTs in native diffusion space. The resulting CST masks are shown in Figure 3b (left/right hemisphere). These masks represent the spatial extent of the CSTs and serve as anatomical references for evaluating motor pathway involvement.

To ensure compatibility with lesion masks, CST outputs were resampled using nearest-neighbour interpolation, preserving their binary structure. For consistency across data types, we extracted the first volume from 4D multi-timepoint DWI acquisitions to match the dimensionality of single-volume lesion segmentations.

#### 3.3.7. CST Overlap Metrics

CST overlap metrics quantified the spatial relationship between the lesion and the CST. The CST was segmented using TractSeg [15]. The precomputed peaks from MRtrix3 were fed into TractSeg to generate binary masks for the left and right CST (Figure 3b). The CST masks were resampled to match the lesion mask resolution. For each side, we computed the overlap volume $V_{\mathrm{left}\;\mathrm{overlap}}$ = voxels in (lesion ∩ left CST) and similarly for the right. The overlap percentages are

(19) $$\begin{array}{c} \text{LeftOverlap\%}=\frac{V_{\mathrm{left}\;\mathrm{overlap}}}{V_{\mathrm{left}\;\mathrm{CST}}}\times 100,\;\text{RightOverlap\%}=\frac{V_{\mathrm{right}\;\mathrm{overlap}}}{V_{\mathrm{right}\;\mathrm{CST}}}\times 100 \end{array}$$

All imaging biomarkers used for functional outcome prediction were derived from lesion masks and CST segmentation, spanning morphological, intensity-based, texture, and anatomical features. A detailed summary of these extracted features and their corresponding formulas is provided in Table 5.

### 3.4. Machine Learning for mRS Prediction

The objective of the outcome modelling component was to investigate whether imaging-derived lesion and tract-based biomarkers, extracted using a fully automated pipeline and routine clinical MRI, are associated with functional outcome at discharge. Given the limited cohort size and the exploratory nature of this analysis, the task was formulated as a binary classification problem, distinguishing between a favourable outcome (mRS ≤ 2) and an unfavourable outcome (mRS > 2) at discharge. This dichotomisation reflects functional independence in daily activities and is widely adopted in clinical stroke research and interventional trials.

**Feature Set Construction and Selection:** For each subject, a total of 19 imaging-derived features were extracted, encompassing complementary descriptors of lesion burden and anatomical involvement. These features included volumetric and morphological lesion measures, quadrant-based spatial distribution, ADC-derived intensity and texture features, and anatomically informed corticospinal tract (CST) overlap metrics. The inclusion of this heterogeneous feature set was motivated by the need to capture multiple aspects of stroke pathology, including lesion size, shape complexity, spatial localisation, tissue heterogeneity, and motor pathway involvement.

To reduce feature redundancy and limit the impact of multicollinearity—particularly important given the modest sample size–univariate ANOVA-based feature selection (SelectKBest) was applied. Feature selection was performed exclusively within the training data to avoid information leakage. The top 17 features with the strongest statistical association to the outcome labels were retained for model training. This step serves as a lightweight dimensionality reduction strategy, preserving interpretability while improving model stability.

**Dataset Composition and Splitting Strategy:** Outcome modelling was conducted exclusively on the ISLES 2024 dataset, as it is the only cohort providing discharge modified Rankin Scale (mRS) annotations. Of the 149 publicly available ISLES 2024 subjects, 52 cases lacked discharge mRS labels and were therefore excluded from outcome modelling. The remaining 97 subjects constituted the final analysis cohort.

These cases were randomly divided into 80% training (n = 77) and 20% held-out testing (n = 20) subsets, with stratification applied to preserve the proportion of favourable (mRS ≤ 2) and unfavourable (mRS > 2) outcomes in both splits. This splitting strategy was chosen to balance model learning capacity with the need for an independent evaluation set, given the limited cohort size.

All preprocessing steps related to feature selection, hyperparameter optimisation, and model training were performed exclusively on the training subset. The held-out test set was used only once, for final performance evaluation, ensuring a strict separation between model development and assessment and minimising the risk of information leakage.

**Model Choice and Training:** Three tree-based ensemble classifiers–Random Forest, XGBoost, and CatBoost were selected for evaluation. These models were chosen for several reasons: (1) their suitability for heterogeneous, tabular feature spaces; (2) their robustness to non-linear feature interactions; and (3) their ability to provide intrinsic measures of feature importance, which is essential for interpretability in clinical contexts.

Model hyperparameters were optimised using grid search combined with five-fold cross-validation, conducted exclusively on the training set. This approach balances model flexibility with overfitting control and is appropriate for small-to-moderate sample sizes.

**Performance Evaluation and Uncertainty Quantification:** Model performance was evaluated on the held-out test subset using standard classification metrics, including accuracy, precision, recall, F1-score, and area under the receiver operating characteristic curve (ROC-AUC). Given the limited size of the test set, uncertainty in performance estimates was explicitly quantified by computing 95% confidence intervals for all primary metrics using resampling-based procedures. This approach provides a more informative assessment than single-point estimates and reflects the sensitivity of performance metrics to small sample variations. No formal hypothesis testing or statistical significance tests were performed to compare model performance (e.g., pairwise AUC comparisons). Accordingly, observed differences between models are reported descriptively and interpreted with caution, with the emphasis placed on robustness, uncertainty, and consistency rather than claims of superiority.

**Interpretability and Feature Importance Analysis:** To enhance transparency and support clinical interpretability, feature importance analyses were conducted for each trained model. These analyses were used to identify imaging-derived biomarkers that contributed most strongly to outcome classification. Importantly, feature importance results are interpreted as associative and hypothesis-generating, rather than causal, and are intended to provide insight into potential structural correlates of functional recovery.

Overall, this modelling framework is designed to evaluate the feasibility and interpretability of imaging-based outcome modelling using routine MRI under realistic data constraints, rather than to establish a definitive predictive system. The results should therefore be interpreted as exploratory and motivating further validation on larger, prospectively collected cohorts.

## 4. Results

### 4.1. Lesion Segmentation Performance

Lesion segmentation performance was evaluated on two distinct datasets described in Section 3.1 corresponding to different experimental settings. ISLES 2022 was used as an internal benchmark for multi-modal lesion segmentation, where DWI, ADC, and FLAIR images were available and the full ensemble framework could be applied. In contrast, ISLES 2024 served as an external evaluation dataset characterised by greater heterogeneity in scanners, acquisition protocols, and preprocessing pipelines, as well as limited modality availability. For ISLES 2024, lesion segmentation was therefore performed exclusively using the SEALS model with the DWI and ADC modalities in a zero-shot setting, without retraining or fine-tuning.

To contextualise the segmentation performance on ISLES 2022, we compared the proposed ensemble framework against representative deep learning–based lesion segmentation approaches reported in the literature. These include conventional 3D U-Net and Attention U-Net architectures trained on multi-modal MRI inputs. The comparison focuses on Dice similarity coefficients reported under comparable multi-modal settings and is intended to provide reference context rather than a direct statistical benchmark, as differences in training data, preprocessing, and evaluation protocols preclude strict quantitative equivalence across studies.

The quantitative segmentation results are summarised in Table 6. On the ISLES 2022 benchmark, where DWI, ADC, and FLAIR modalities were available, the proposed ensemble framework achieved a mean Dice similarity coefficient of 0.82. Under the same experimental conditions, the individual constituent models achieved Dice scores of 0.81 (SEALS), 0.82 (NVAUTO), and 0.76 (FACTORIZER), indicating that voxel-wise majority voting yields slightly more stable performance than single-model segmentation. Table 6 also reports Dice similarity coefficients from representative deep learning–based lesion segmentation methods published in the literature. Compared to these studies, which report Dice values of 0.78 for an Attention U-Net [38], 0.774 for a multi-modal 3D U-Net [39], and 0.75 for a 3D U-Net trained on DWI [40], the Dice score obtained by the proposed ensemble on ISLES 2022 is numerically higher by approximately 2–7%. These numerical differences are reported for contextual reference only, as variations in datasets, imaging modalities, preprocessing pipelines, and evaluation protocols preclude direct statistical comparison or claims of superiority.

**Figure 4:**
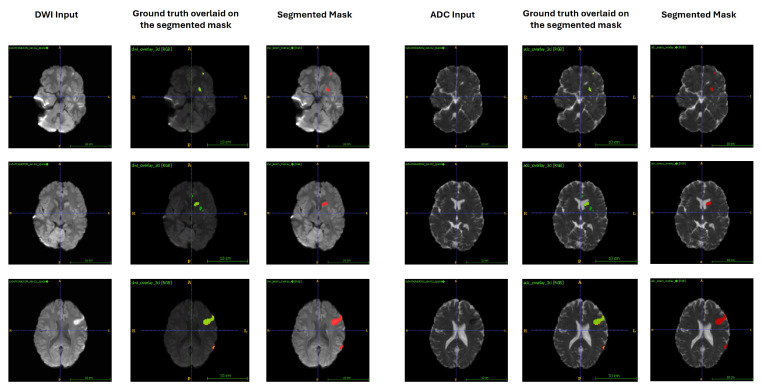
Qualitative lesion segmentation results from the ISLES 2024 dataset. For each case, DWI and ADC input images are shown alongside predicted lesion masks (red) and ground-truth annotations (green). Three representative axial slices illustrate small, medium, and large infarcts. The examples demonstrate spatial correspondence between predicted and reference masks under zero-shot external evaluation conditions, with discrepancies primarily observed at lesion boundaries.

To complement the quantitative evaluation, Figure 4 presents representative qualitative segmentation examples from the ISLES 2024 dataset. Predicted lesion masks (red) and ground-truth annotations (green) are overlaid on both DWI and ADC images for cases with small, medium, and large infarcts. The examples demonstrate consistent localisation of ischemic regions across lesion sizes, with discrepancies primarily observed at lesion boundaries rather than large-scale mislocalisation. These qualitative results support the use of the automated segmentations for downstream feature extraction, while also illustrating the uncertainty inherent to zero-shot external deployment.

Overall, the segmentation results indicate that the proposed framework provides sufficiently accurate and spatially consistent lesion masks under both benchmark and external conditions, forming a reliable basis for the extraction of imaging biomarkers used in subsequent exploratory outcome modelling.

### 4.2. External Validation

External validation of the lesion segmentation framework was conducted using the ISLES 2024 dataset, which differs substantially from ISLES 2022 in terms of scanner vendors, acquisition protocols, preprocessing pipelines, and modality availability. While the ensemble framework was trained using DWI, ADC, and FLAIR modalities, only the SEALS model is compatible with the DWI and ADC sequences consistently available in ISLES 2024. As a result, it was not feasible to evaluate the full ensemble or the other constituent models on this dataset.

Segmentation on ISLES 2024 was therefore performed using the SEALS model alone in a zero-shot configuration, without retraining or fine-tuning. Under these conditions, a mean Dice similarity coefficient of 0.57 was obtained. This reduction in performance relative to ISLES 2022 reflects the combined effects of reduced modality availability, the absence of ensemble voting, and the increased heterogeneity of the external dataset, rather than a failure of the underlying segmentation approach.

These results highlight the challenges associated with deploying lesion segmentation models across heterogeneous clinical settings and emphasise the importance of external validation under realistic constraints. In this work, the external evaluation serves to characterise the expected behaviour of the segmentation pipeline in out-of-distribution scenarios and to inform the interpretation of downstream feature extraction and outcome modelling.

### 4.3. CST Overlap and Outcome Association

To characterise the involvement of motor pathways, we quantified the spatial overlap between the segmented ischemic lesion masks and CST, which was delineated using TractSeg. The CST is a principal pathway for voluntary motor control and is commonly affected in ischemic stroke due to its compact anatomical course through regions such as the internal capsule and corona radiata. The lesion–CST overlap metric captures anatomical information that is not conveyed by lesion volume alone, as lesions of similar size may differ substantially in their functional impact depending on their spatial relationship to critical white matter tracts. In this work, overlap was computed separately for the left and right CST and expressed as the proportion of CST voxels intersecting with the lesion mask.

**Figure 5:**
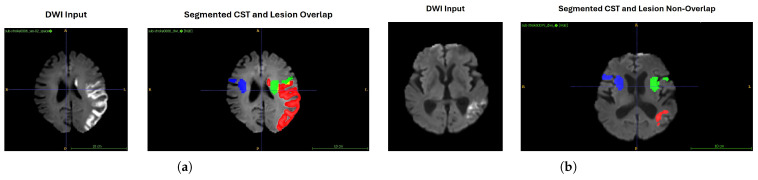
Visualisation of lesion–CST overlap and non-overlap on ISLES 2024 dataset cases. (**a**) The patient with an unfavourable outcome (mRS > 2) on DWI modality (**b**) The patient with a favourable outcome (mRS < 2) on DWI modality. The ischemic lesion (red) and CST segmentation (green) are overlaid; yellow voxels represent areas of intersection, highlighting damage to the motor pathway. This overlap is used to quantify CST involvement and is significantly associated with unfavourable functional outcomes (mRS). In comparison, the cases with no overlap have favourable functional outcomes (mRS). Orientation labels and scale bars are shown for anatomical reference.

Figure 5 illustrates representative examples from the ISLES 2024 dataset, highlighting cases with lesion–CST overlap and non-overlap. In the example shown in Figure 5a, the ischemic lesion intersects with the CST, whereas in Figure 5b, the lesion is spatially separated from the tract. These visualisations are provided to demonstrate how the overlap metric captures anatomically meaningful differences in lesion location relative to motor pathways.

Across the analysed cohort, the cases of ISLES 2022 and 2024 with unfavourable functional outcomes at discharge (mRS > 2) tended to exhibit greater lesion–CST overlap than cases with favourable outcomes (mRS ≤ 2). This observation is consistent with established neuroanatomical knowledge linking CST involvement to motor impairment. In the present study, lesion–CST overlap is therefore treated as an anatomically informed imaging feature and incorporated into the exploratory outcome modelling framework, rather than as an independent predictor of disability.

### 4.4. Functional Outcome Prediction

Functional outcome prediction was evaluated using imaging-derived biomarkers extracted from the ISLES 2024 dataset. Three tree-based machine learning classifiers, Random Forest, CatBoost, and XGBoost were trained to predict binary modified Rankin Scale (mRS) outcomes at discharge (favourable vs. unfavourable). All models were trained on the same feature set, incorporating lesion volume, morphological descriptors, ADC-based texture features, quadrant-based spatial information, and corticospinal tract (CST) overlap metrics.

**Figure 6:**
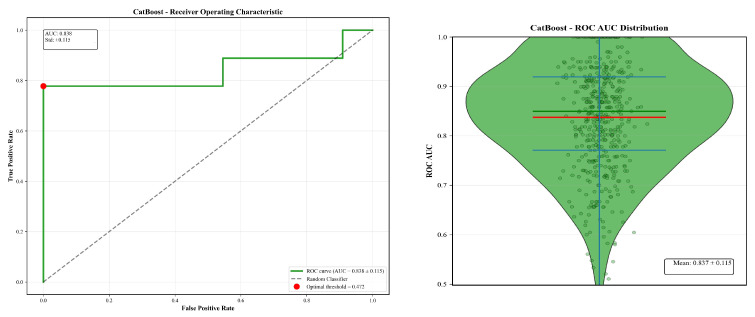
ROC and bootstrap AUC results for the CatBoost model on a small test set (n = 20). The ROC plot shows the characteristic step pattern that appears when every individual case influences the curve. The accompanying AUC distribution illustrates how performance varies when the test data are repeatedly resampled, with most values clustering around the mid-0.8 range. Together, these visuals highlight both the model’s strong discriminative ability and the uncertainty introduced by the limited sample size.

**Quantitative Performance Evaluation:** The quantitative performance of the three classifiers on the held-out test set (n = 20) is summarised in Table 7. Confusion matrices for all three classifiers are provided in Supplementary Figures S1–S3. Across all metrics, the models exhibited comparable performance, with overlapping confidence intervals reflecting the uncertainty associated with the limited test sample size. Bootstrap-derived confidence intervals for all performance metrics are reported in Supplementary Figures S4–S6. CatBoost achieved an accuracy of 0.88 (95% CI: 0.60–0.90) and an F1-score of 0.87 (95% CI: 0.60–0.90), while Random Forest and XGBoost yielded similar F1-scores (0.80) and ROC-AUC values (0.82 and 0.81, respectively). Given the small test set, performance differences between models are reported descriptively, and no claims of statistical superiority are made. The results indicate that all three models are capable of leveraging the extracted imaging features for outcome discrimination under the evaluated conditions. ROC Analysis and Uncertainty Quantification: To further characterise model behaviour under uncertainty, Figure 6 presents the receiver operating characteristic (ROC) curve and bootstrap AUC distribution for the CatBoost classifier, shown here as a representative example. ROC curves for Random Forest and XGBoost are provided in Supplementary Figures S7 and S8. The ROC curve exhibits a step-wise pattern, reflecting the influence of individual test cases in a small evaluation cohort. The accompanying bootstrap AUC distribution illustrates the variability of AUC estimates under repeated resampling of the test set.

Rather than providing a single point estimate, the bootstrap distribution highlights the range of plausible performance values and underscores the uncertainty inherent in a small-sample evaluation. The central tendency of the distribution remains consistent with the reported ROC-AUC value, supporting the internal consistency of the evaluation while avoiding over-interpretation.

**Comparison with Prior Imaging-Based Outcome Models:** For contextual reference, Table 8 summarises the performance of the proposed imaging-based framework alongside previously reported MRI-based models for predicting discharge modified Rankin Scale (mRS) outcomes. In this study, the CatBoost classifier achieved an accuracy of 0.88 and an F1-score of 0.87 on the ISLES 2024 dataset using a feature set that integrates lesion morphology, spatial localisation, ADC-derived texture descriptors, and corticospinal tract (CST) involvement.

By comparison, Wei et al. [21] reported an accuracy of 0.77 and an F1-score of 0.76 using logistic regression trained on DWI and ADC radiomic features, while Yu et al. [41] achieved an accuracy of 0.83 and an F1-score of 0.78 using a LightGBM model trained on multi-modal radiomics (DWI, ADC, FLAIR, SWI, and T1-weighted MRI). Relative to these studies, the performance metrics observed in the present work are numerically higher, with differences of approximately 5–11% in accuracy and 9–11% in F1-score.

These numerical differences should be interpreted with caution, as the studies differ in cohort composition, imaging modalities, feature definitions, outcome timing, and evaluation protocols. Accordingly, Table 8 is intended to provide contextual positioning of the proposed framework within the existing literature rather than to support direct quantitative comparison or claims of methodological superiority.

**Figure 7:**
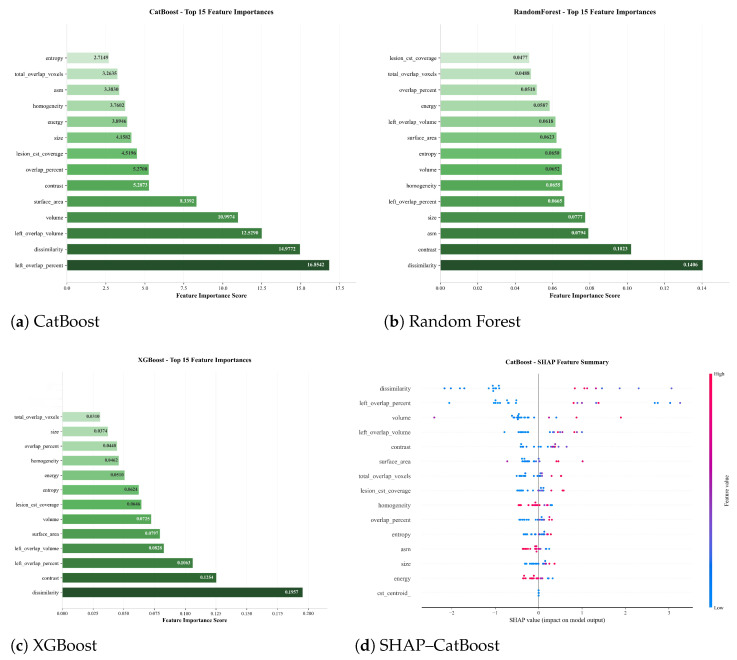
Visualisation of the top predictive imaging features extracted from the ISLES 2024 dataset across models used for mRS prediction at discharge. (**a**) CatBoost model: overlap percentage of lesion–CST, dissimilarity, volume, surface area, and contrast as dominant predictors. (**b**) Random Forest: highlights dissimilarity, contrast, ASM, size homogeneity, and lesion–CST overlap. (**c**) XGBoost: emphasises dissimilarity, contrast, lesion–CST overlap, surface area, and volume. (**d**) SHAP feature importance for CatBoost, illustrating each feature’s contribution to mRS prediction. All models consistently identify lesion-derived features as key determinants of functional outcome.

**Feature Importance and Model Interpretability:** The relative importance of imaging-derived features contributing to functional outcome prediction is illustrated in Figure 7 for all three classifiers, with a SHAP-based explanation provided for the CatBoost model. Across Random Forest, XGBoost, and CatBoost, a consistent pattern emerges in which features capturing lesion extent, spatial involvement of the corticospinal tract (CST), and intra-lesional texture heterogeneity rank among the most influential. The feature importance rankings for all three models are provided in Supplementary Figures S9–S11. Among anatomical features, lesion–CST overlap metrics (including overlap percentage and overlap volume) consistently appear among the top-ranked predictors across all models. This indicates that the spatial relationship between the infarct and motor pathways provides information beyond lesion size alone and is frequently utilised by the classifiers during decision-making. From a neuroanatomical perspective, this aligns with the known role of CST integrity in motor recovery and functional independence.

Measures of lesion extent and morphology, including lesion volume and surface area, also rank highly across models. Larger and more spatially extensive lesions are associated with a higher likelihood of unfavourable outcomes, reflecting the cumulative burden of tissue damage. The prominence of surface area alongside volume suggests that lesion shape complexity, not only absolute size, contributes to outcome discrimination.

ADC-based texture features, particularly dissimilarity and contrast, are repeatedly identified as influential. These features quantify local intensity variability within the lesion and may capture heterogeneity related to infarct composition, partial tissue damage, or mixed diffusion patterns. In the SHAP summary plot for CatBoost (Figure 7d), higher values of dissimilarity and contrast tend to be associated with shifts in the model output toward unfavourable outcomes, whereas lower values contribute to predictions of favourable recovery. This directional behaviour highlights how texture-derived descriptors influence the model’s decisions in a consistent manner.

The SHAP analysis further illustrates that the contribution of individual features is not uniform across subjects. Instead, feature effects vary depending on their value range and interaction with other lesion characteristics, underscoring the non-linear nature of the learned decision boundaries. Importantly, no single feature dominates the prediction; rather, outcome discrimination emerges from the combined contribution of lesion size, anatomical location relative to motor pathways, and microstructural heterogeneity.

Feature importance results are interpreted as associative and hypothesis-generating, rather than causal. Their primary purpose is to enhance transparency and clinical interpretability by identifying which imaging-derived characteristics are most frequently leveraged by the models during prediction. These findings motivate further investigation into the combined role of lesion topology and tissue heterogeneity in functional recovery, ideally in larger, prospectively collected cohorts with richer clinical annotation.

Overall, these results demonstrate the feasibility of combining automated lesion segmentation, tract-based anatomical features, and machine learning for exploratory prediction of functional outcome using routine clinical MRI. The findings motivate further validation on larger, prospectively collected cohorts before clinical deployment.

Collectively, these results highlight the significant prognostic value of combining lesion segmentation, tract-based anatomical biomarkers, and machine learning for outcome prediction. The model demonstrates clinical interpretability and substantial improvement over baseline imaging-only or clinical-only approaches.

## 5. Discussion

This study presents a fully automated MRI-based pipeline for ischemic stroke lesion analysis and functional outcome prediction, designed to operate on routine clinical imaging. Under matched training and evaluation conditions, the lesion segmentation component achieved a Dice similarity coefficient of 0.82 on the ISLES 2022 test set, consistent with state-of-the-art performance for multi-modal stroke lesion segmentation. When applied to the ISLES 2024 cohort in a zero-shot configuration, segmentation performance decreased to a Dice score of 0.57, reflecting the combined effects of domain shift, reduced modality availability, and increased heterogeneity in acquisition protocols and preprocessing pipelines.

These external results should be interpreted as an assessment of feasibility under realistic deployment constraints, rather than as evidence of clinical readiness. The ISLES 2024 evaluation intentionally reflects a challenging scenario in which models trained under controlled conditions are applied to unseen data without adaptation. While domain adaptation, fine-tuning, or modality-harmonisation strategies could reasonably be expected to improve performance, such optimisation was beyond the scope of the present work. Further validation on larger and more diverse cohorts will be required to establish robustness across clinical settings.

A key strength of the proposed framework lies in its focus on anatomically meaningful and interpretable imaging biomarkers, particularly those related to corticospinal tract (CST) involvement. By combining automated lesion segmentation with TractSeg-derived CST maps, we were able to quantify lesion–tract overlap as a spatially informed measure of motor pathway disruption. This feature consistently ranked among the most influential predictors of functional outcome and provides a more anatomically grounded indicator of motor impairment than lesion volume or location alone. These findings align with longstanding clinical and neuroanatomical evidence linking CST integrity to post-stroke motor recovery.

In the outcome prediction task, CatBoost achieved the highest point estimates (accuracy 0.88, F1-score 0.87) among the evaluated classifiers. However, performance differences between CatBoost, Random Forest, and XGBoost were not statistically significant, and all results should be interpreted cautiously given the small size of the held-out test set. Rather than claiming model superiority, these findings demonstrate that multiple tree-based ensemble methods are capable of leveraging the extracted imaging features for outcome discrimination under exploratory conditions. Compared to prior imaging-based frameworks that rely primarily on volumetric or radiomic features, the present feature set integrates lesion morphology, spatial localisation, texture heterogeneity, and CST involvement, offering a richer and more biologically grounded representation of stroke pathology.

Importantly, the proposed models were intentionally restricted to imaging-derived features in order to isolate the added predictive value of lesion characteristics. Established clinical predictors such as age, baseline NIHSS, and time to imaging were excluded to reduce collinearity and mitigate overfitting in a limited sample. This design choice does not imply that imaging alone is sufficient for clinical decision-making. Rather, the imaging-based biomarkers presented here are intended to complement, not replace, existing clinical assessments. Integrating clinical and imaging features in future studies may further improve predictive performance and clinical utility, provided adequate sample sizes are available.

Clinical feasibility represents another advantage of the framework. All inputs are derived from routinely acquired DWI and ADC sequences, without the need for advanced diffusion protocols, manual intervention, or specialised hardware. The pipeline is fully automated and achieved end-to-end processing times of approximately 2–3 min per case, which is compatible with time-sensitive acute stroke workflows. Nevertheless, real-world deployment will require prospective evaluation to assess integration into clinical systems, robustness under variable computational resources, and impact on workflow efficiency. Approaches such as model optimisation or cloud-based processing may help mitigate infrastructure limitations in resource-constrained settings.

Feature analysis further highlighted the prognostic relevance of interpretable lesion biomarkers. Lesion volume, surface area, contrast, texture dissimilarity, and CST overlap consistently emerged as dominant predictors, each capturing complementary aspects of infarct burden and motor system involvement. Together, these features describe lesion extent, boundary complexity, tissue heterogeneity, and anatomical disruption of critical motor pathways. While these associations are biologically plausible, feature importance results are interpreted as associative and hypothesis-generating, rather than causal, and should be validated in larger cohorts.

Several limitations warrant consideration. Firstly, CST segmentation was performed automatically using TractSeg applied to single-shell DWI, which may be less accurate than multi-shell diffusion or expert-curated tractography. The absence of manual CST annotations prevents direct validation of tract localisation accuracy, and lesion–CST overlap measures should therefore be interpreted cautiously. Secondly, the domain shift between ISLES 2022 and ISLES 2024 highlights the challenge of generalisation across heterogeneous datasets, particularly under zero-shot conditions. Thirdly, although baseline demographic and clinical characteristics were reported, they were not incorporated into the predictive models. Fourthly, dichotomisation of mRS into favourable (≤2) and unfavourable (>2) outcomes, while common in prognostic studies, reduces outcome granularity and may obscure clinically meaningful distinctions.

## 6. Conclusions and Future Works

This work proposes a fully automated, imaging-based framework for exploring functional outcome prediction in acute ischemic stroke using routinely acquired MRI. By integrating lesion segmentation, anatomically informed corticospinal tract (CST) overlap analysis, and machine learning–based modelling, the study demonstrates the technical feasibility of extracting interpretable imaging biomarkers that capture lesion extent, spatial localisation, and tissue heterogeneity without relying on advanced diffusion protocols or manual intervention.

The results support the hypothesis that combining lesion morphology, ADC-derived texture descriptors, and tract-based anatomical context provides complementary prognostic information beyond lesion volume alone. While outcome prediction performance is encouraging, the present findings should be regarded as exploratory and hypothesis-generating, given the limited sample size and the challenges associated with external, zero-shot evaluation.

Future work will focus on strengthening robustness and clinical relevance through validation on larger, multi-centre cohorts and by reducing domain shift via harmonisation or domain-adaptive learning strategies. Integrating clinical variables such as age, baseline NIHSS, time from onset, and perfusion imaging is expected to further enhance predictive performance and clinical utility. Methodologically, improvements in lesion and tract segmentation potentially through self-supervised learning, transformer-based architectures, or uncertainty-aware modelling, may reduce error propagation and improve reliability.

Finally, prospective evaluation within real-world stroke workflows, including assessment of computational efficiency, interpretability, and clinical impact, will be essential to determine whether anatomically informed imaging biomarkers can meaningfully support personalised prognosis and rehabilitation planning in routine care.

## Figures and Tables

**Table 2:** Comparison of imaging specifications between ISLES 2022 and ISLES 2024 datasets. The substantial heterogeneity in scanner hardware, acquisition protocols, and voxel geometry across datasets reflects real-world clinical variability and enables assessment of segmentation robustness under external, zero-shot evaluation conditions.

Specification	ISLES 2022	ISLES 2024
**Source**	Multicentre (3 European hospitals: Munich, Bern, Hamburg)	Multicentre (large-vessel occlusion ischemic stroke cohorts across multiple centres)
**Number of Cases**	400 (250 training, 150 test)	249 (149 training, 100 test (not available publicly))
**Image Modalities**	FLAIR, DWI (b = 1000 s/mm^2^), ADC	NCCT, CTA, CTP (with perfusion maps: Tmax/CBF/CBV/MTT), follow-up DWI/ADC (2–9 days)
**Preprocessing**	Skull-stripped, co-registered, NIfTI format; released in native space after skull stripping	Raw and pre-processed formats both available; organised in BIDS; defaced scans; registered to NCCT space for modalities where applicable
**FLAIR Resolution**	In-plane: 0.23 × 0.23 mm^2^ to 2 × 2 mm^2^ Slice thickness: 0.7–9.6 mm Volume: 112 × 112 × 72 voxels	Not available
**DWI/ADC** **Resolution**	In-plane: 0.88 × 0.88 mm^2^ to 2 × 2 mm^2^ Slice thickness: 2–6.5 mm Resampled to 2 × 2 × 2 mm^3^	In-plane: 0.410×0.410 mm^2^ Slice thickness: 2.0 mm Volume: 512×633×70 voxels

**Table 3:** Clinical and demographic characteristics of the ISLES 2024 cohort (n = 149).

Characteristic	Value
Age (years), mean ± SD	71.9 ± 14.4
Sex (male/female)	44%/52%
Baseline NIHSS	11.5 ± 6.2
Time to MRI (days)	2–9
mRS at 24 h	3.4 ± 1.5
Favourable outcome (mRS ≤2)	47%
Unfavourable outcome (mRS >2)	53%

**Table 4:** MRtrix3 preprocessing and TractSeg segmentation commands. DWI data were converted to MRtrix format, with reference peaks transformed into the DWI space, and converted to NIfTI. The corticospinal tracts (CST) were then segmented using the pre-trained TractSeg model on the preprocessed data.

Processing Step	Command
**MRtrix3 Preprocessing**	
Convert DWI to MRtrix format	mrconvert dwi_ax1.nii.gz dwi_ax.mif
Convert reference peaks to MRtrix format	mrconvert peaks_ref.nii.gz ref_peaks_3D.mif
Transform peaks to match DWI space	mrtransform ref_peaks_3D.mif peaks_for_data_3D.mif -template dwi_ax.mif
Convert transformed peaks to NIfTI	mrconvert peaks_for_data_3D.mif peaks_T1.nii.gz
**TractSeg Segmentation**	
Segment corticospinal tracts from preprocessed DWI	TractSeg -i dwi_ax1.nii.gz -o ./ - -raw_diffusion_input

**Table 5:** Summary of extracted features from MRI for outcome prediction. Features are grouped by category and derived from lesion masks and CST segmentations. The formulas are referenced with each description.

Category	Feature	Description/Formula
Morphological	Lesion Volume	Three-dimensional lesion extent using Formula (5)
	Surface Area	Computed using marching cubes algorithm on lesion mesh Equation (7)
	Sphericity	Deviation from spherical geometry Equation (8)
	Solidity	Ratio of lesion volume to convex hull volume Equation (9)
	Elongation	Principal axes length ratio Equation (10)
	Compactness	Volume-to-surface area ratio Equation (11)
Intensity-based	Maximum Intensity	Peak signal intensity Equation (4)
	Average Intensity	Mean signal intensity Equation (5)
	Lesion Centroid	Mass centre coordinates Equation (3)
	Quadrant Distribution	Hemispheric distribution in quadrants Equations (1) and (2)
Texture (GLCM)	Contrast	Local intensity variation Equation (12)
	Dissimilarity	Pairwise pixel differences Equation (13)
	Homogeneity	Regional uniformity degree Equation (14)
	ASM	Grey-level uniformity Equation (15)
	Energy	Pattern regularity metric Equation (16)
	Correlation	Linear dependency strength Equation (17)
	Entropy	Signal randomness measure Equation (18)
Anatomical/Tract-Based	CST Left/Right Volume	Volume of left/right CST masks from TractSeg
	CST Overlap	Hemispheric overlap percentage of Left/Right CST Equation (19)

**Table 6:** Reported Dice similarity coefficients for ischemic stroke lesion segmentation on ISLES 2022 and related multi-modal MRI datasets. Results are shown for contextual comparison only, as methodological differences across studies limit direct quantitative comparison.

Study	Dataset	Method	Dice
Proposed Ensemble segmentation	ISLES 2022	Ensemble	0.82
		SEALS	0.81
		NVAUTO	0.82
		FACTORIZER	0.76
Garcia et al., 2024 [38]	ISLES 2015, 2022	Attention U-Net on multimodal MRI	0.78
Moon et al., 2022 [39]	Multi-centre MRI (FLAIR + DWI)	Multi-Modal 3D U-Net	0.774
Liu et al., 2021 [40]	DWI private cohort	3D U-Net	0.75

**Table 7:** Comparison of ML algorithms’ performance on the ISLES 2024 dataset in predicting modified Rankin Scale (mRS) outcomes at discharge using imaging biomarkers. Three models were trained, and the CatBoost performed better than others.

Model	Accuracy	F1-Score	AUC	MCC	ROC AUC
CatBoost	**0.88 ± 0.17 (CI: 0.6–0.9)**	**0.87 ± 0.17 (CI: 0.6–0.9)**	**0.83 ± 0.16**	**0.8**	**0.83**
Random Forest	0.83 ± 0.08 (CI: 0.6–0.94)	0.80 ± 0.12 (CI: 0.6–0.94)	0.82 ± 0.11	0.7	0.82
XGBoost	0.83 ± 0.11 (CI: 0.6–0.94)	0.80 ± 0.12 (CI: 0.6–0.94)	0.81 ± 0.11	0.7	0.81

**Table 8:** ML algorithms’ performance of MRI-based models in predicting modified Rankin Scale (mRS) outcomes at discharge using imaging biomarkers from the ISLES 2024 dataset. The CatBoost classifier, trained on lesion morphology, CST involvement, and radiomic features, achieved 0.88 accuracy and an F1-score of 0.87.

Study	Features	Model	Accuracy	F1-Score
This work	Lesion + CST + Texture + Morphological	CatBoost	**0.88 ± 0.17**	**0.87 ± 0.17**
Wei et al., 2024 [21]	DWI + ADC radiomics	Logistic Regression	0.77	0.76
Yu et al., 2022 [41]	DWI, ADC, FLAIR, SWI, T1w radiomics	LightGBM	0.83	0.78

## Data Availability

The ISLES 2022 stroke MRI dataset (250 cases with DWI, ADC, and FLAIR scans) is publicly available [32] and was used for training and benchmarking our lesion segmentation. The ISLES 2024 dataset is also publicly available. The code is available in our GitHub repository (https://github.com/mathsholic/Automated-Multi-modal-MRI-Segmentation-of-Stroke-Lesions-and-CST-for-Functional-Outcome-Prediction.git, accessed on 7 September 2025).

## Supplementary Materials

The following supporting information can be downloaded at: https://www.mdpi.com/article/10.3390/tomography12030029/s1.

## Abbreviations

The following abbreviations are used in this manuscript:

AI	Artificial Intelligence
AIS	Acute Ischemic Stroke
ADC	Apparent Diffusion Coefficient
ASM	Angular Second Moment (from GLCM features)
AUC	Area Under the Curve
CNN	Convolutional Neural Network
CSD	Constrained Spherical Deconvolution
CST	Corticospinal Tract
DTI	Diffusion Tensor Imaging
DWI	Diffusion-Weighted Imaging
FACTORIZER	Factorizer Segmentation Model
FLAIR	Fluid-Attenuated Inversion Recovery
FOD	Fibre Orientation Distribution
GLCM	Grey-Level Co-occurrence Matrix
ISLES 2022	Ischemic Stroke Lesion Segmentation (challenge dataset 2022)
ML	Machine Learning
MIF	MRtrix Image Format
MRI	Magnetic Resonance Imaging
mRS	Modified Rankin Scale
NIHSS	National Institutes of Health Stroke Scale
NVAUTO	NVauto Segmentation Model
RF	Random Forest
SA	Surface Area
SEALS	SEALS Segmentation Model
SVM	Support Vector Machine
SWI	Susceptibility-Weighted Imaging
WMH	White Matter Hyperintensities
