# Supplementary figures and images for: Automated Multi-Modal MRI Segmentation of Stroke Lesions and Corticospinal Tract Integrity for Functional Outcome Prediction
Source: Tomography. 2026 Feb 24;12(3):29. doi: 10.3390/tomography12030029 (PMC13030278; doi:10.3390/tomography12030029)

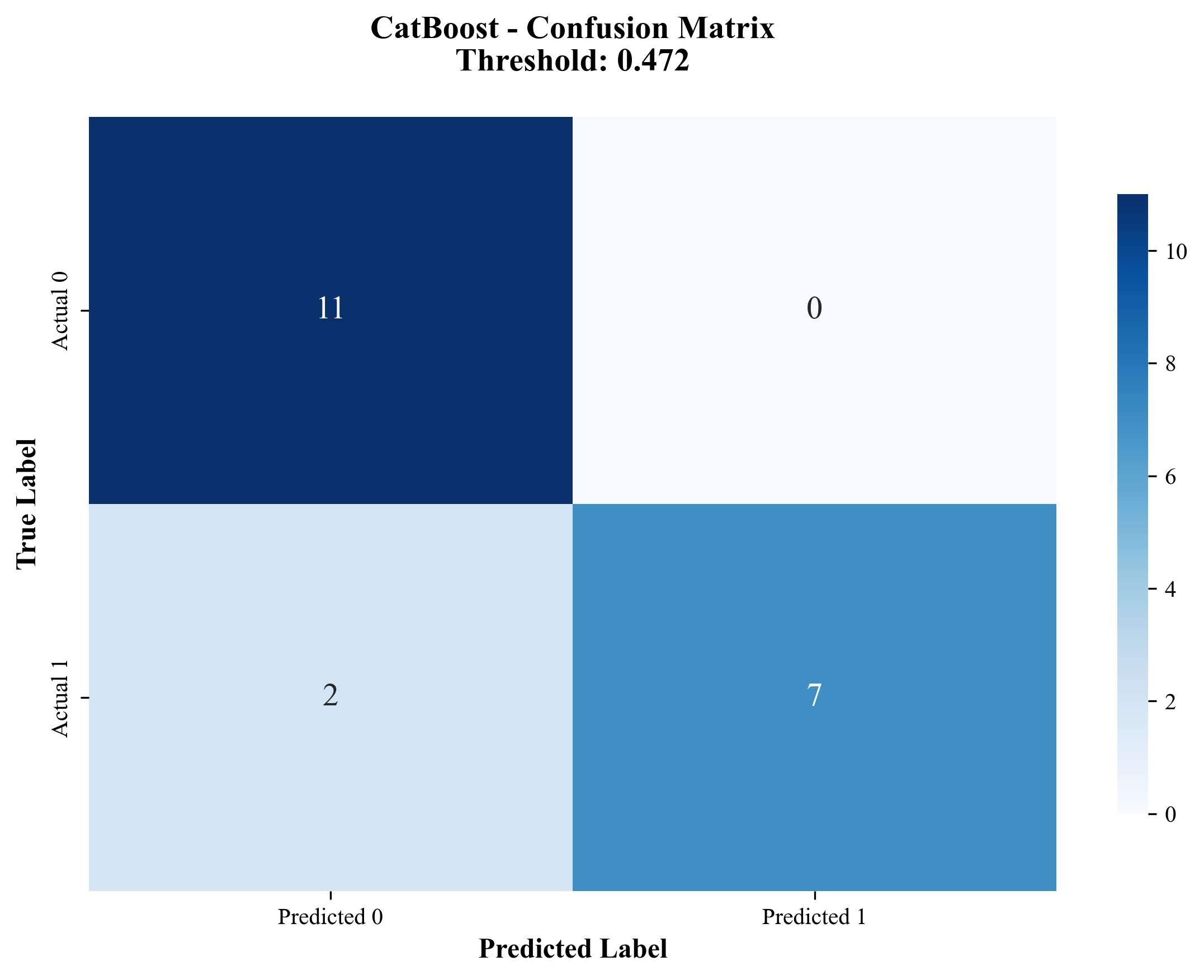

Supplement: Supplementary file 1 [file tomography-12-00029-s001.zip › Figure S1.png]

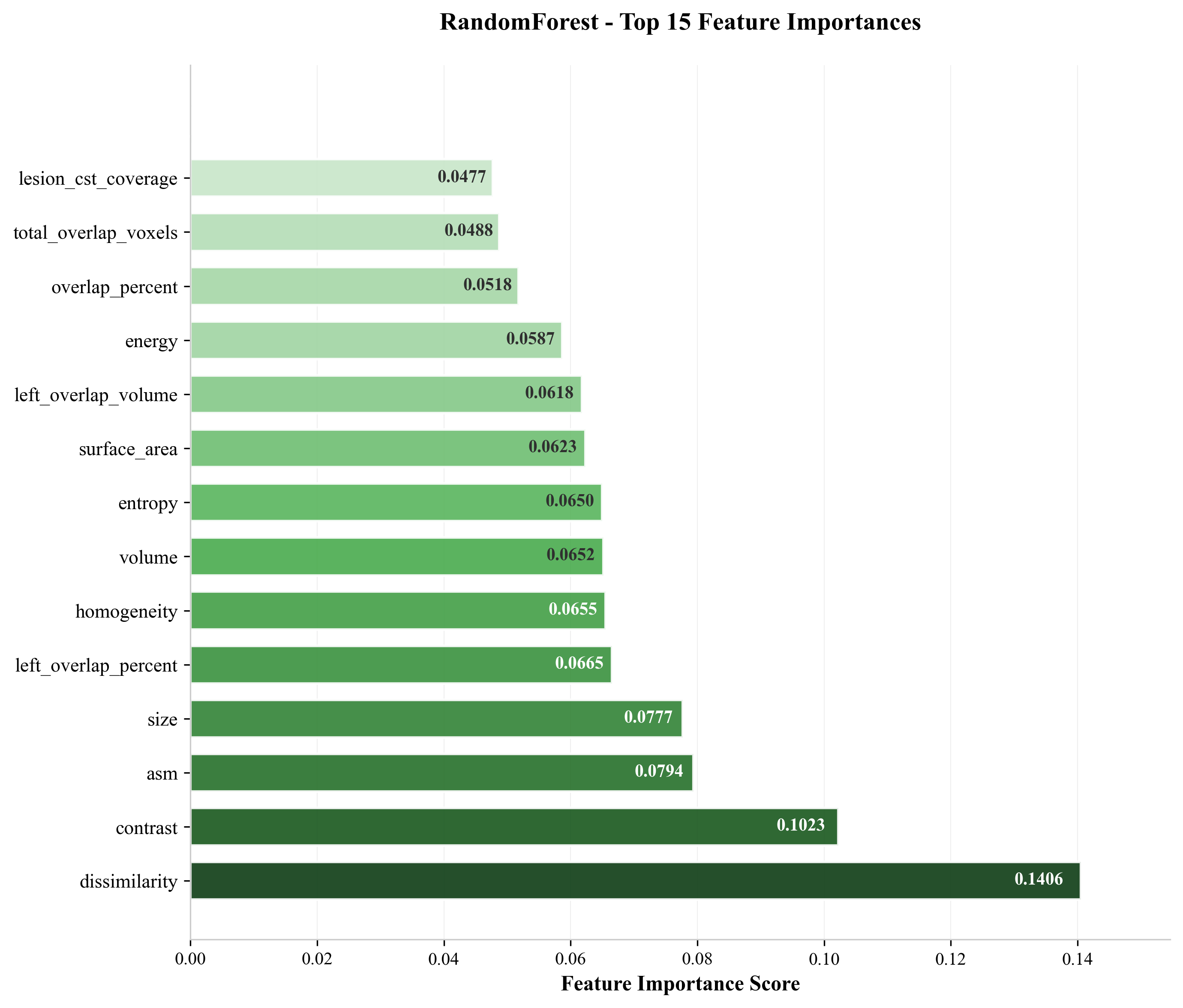

Supplement: Supplementary file 1 [file tomography-12-00029-s001.zip › Figure S10.png]

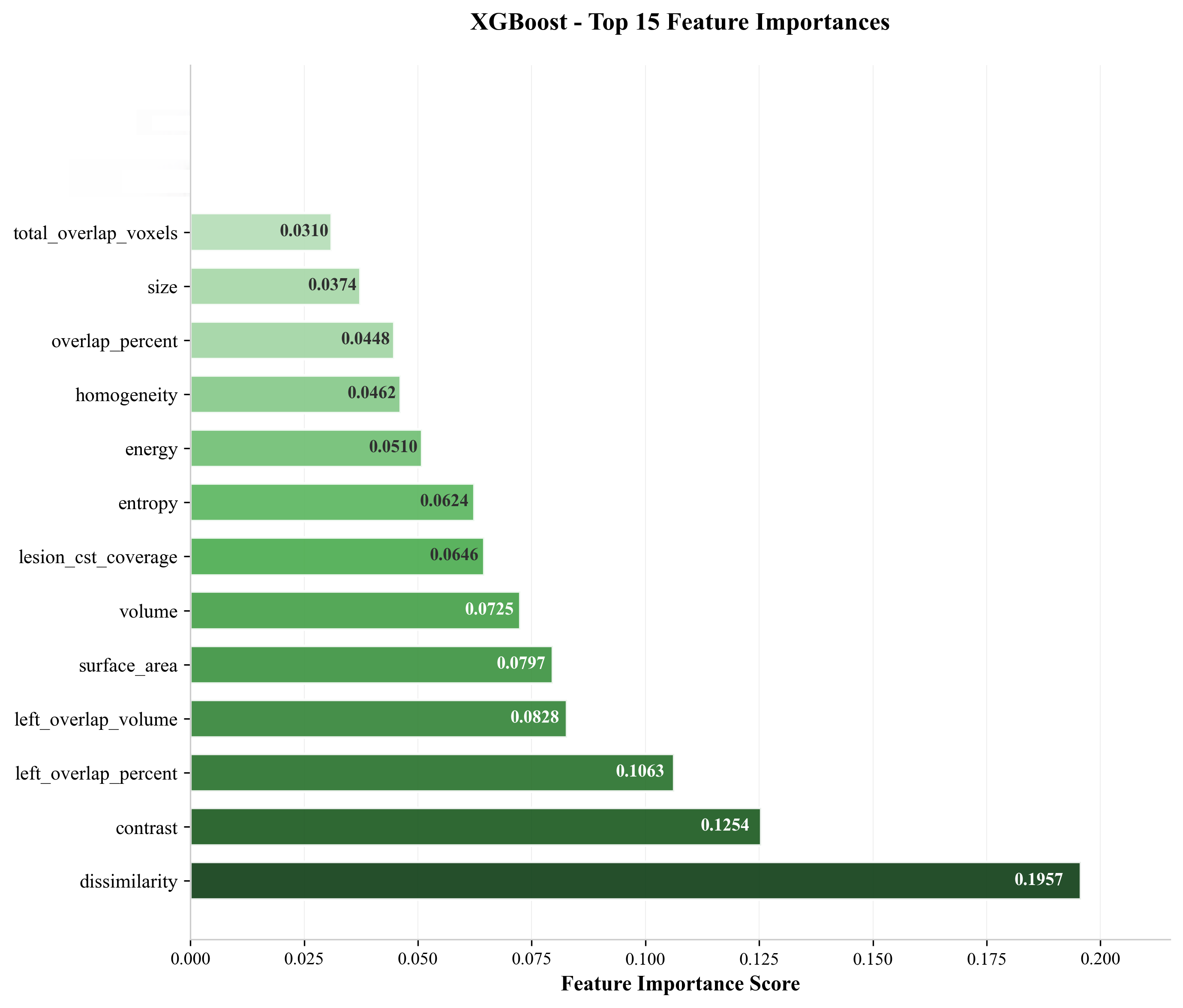

Supplement: Supplementary file 1 [file tomography-12-00029-s001.zip › Figure S11.png]

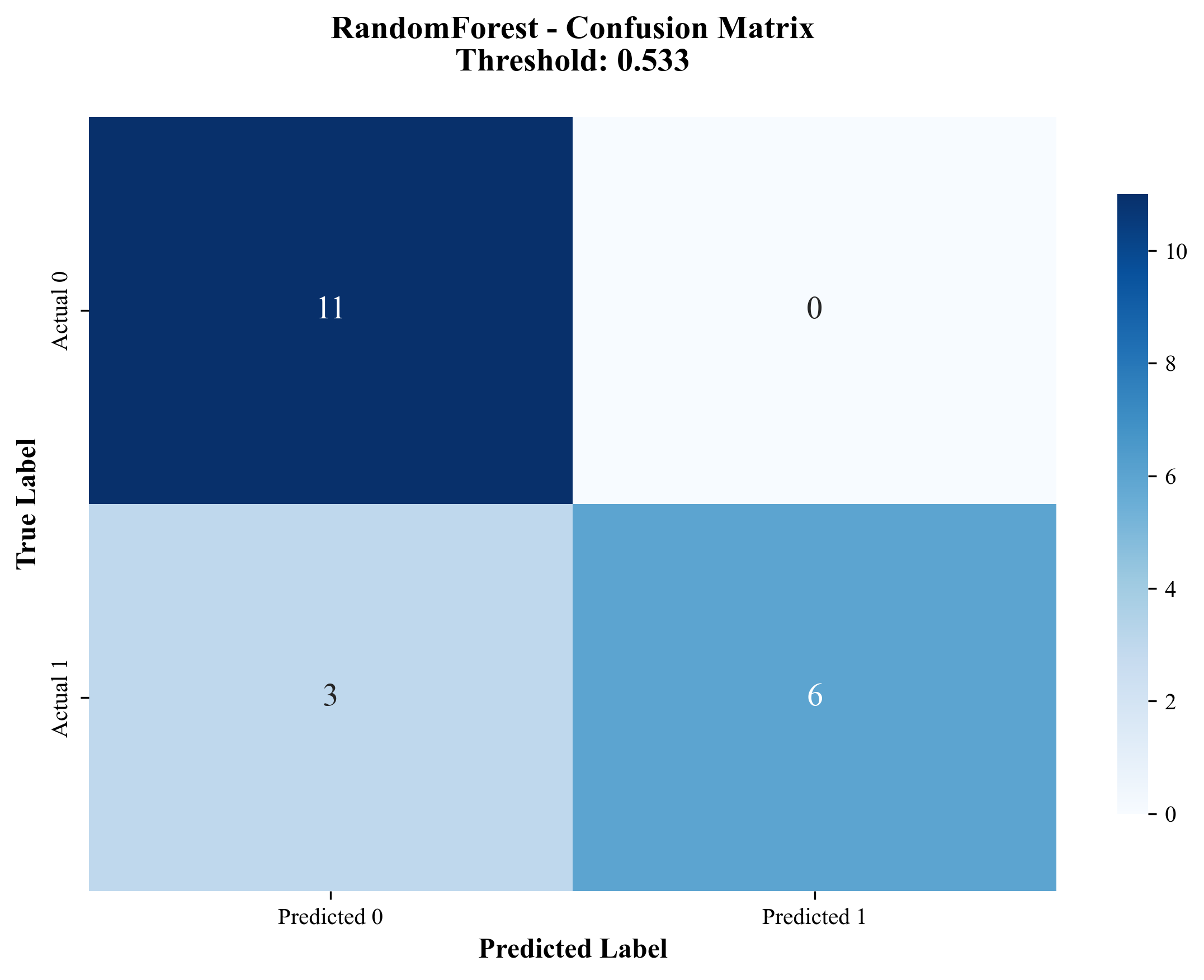

Supplement: Supplementary file 1 [file tomography-12-00029-s001.zip › Figure S2.png]

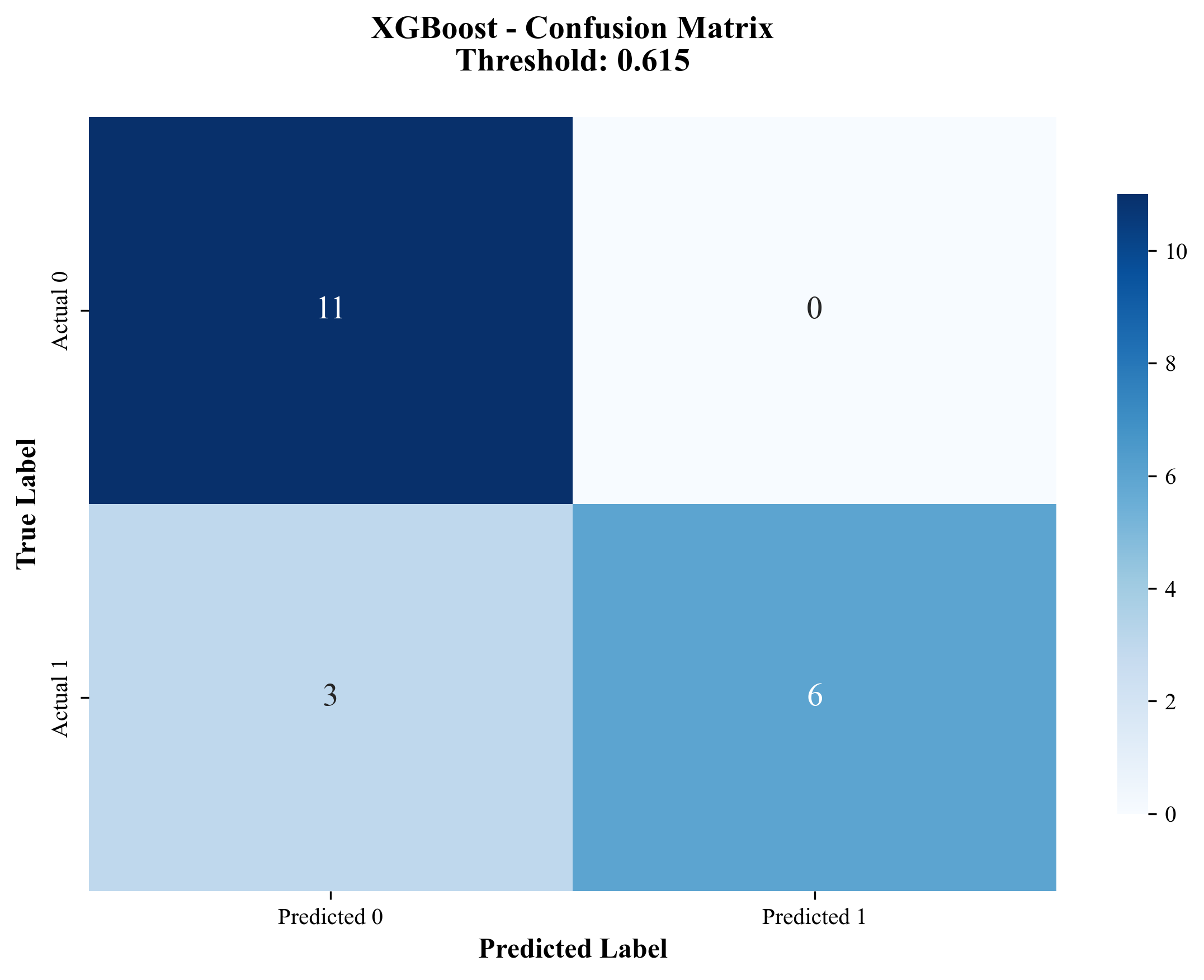

Supplement: Supplementary file 1 [file tomography-12-00029-s001.zip › Figure S3.png]

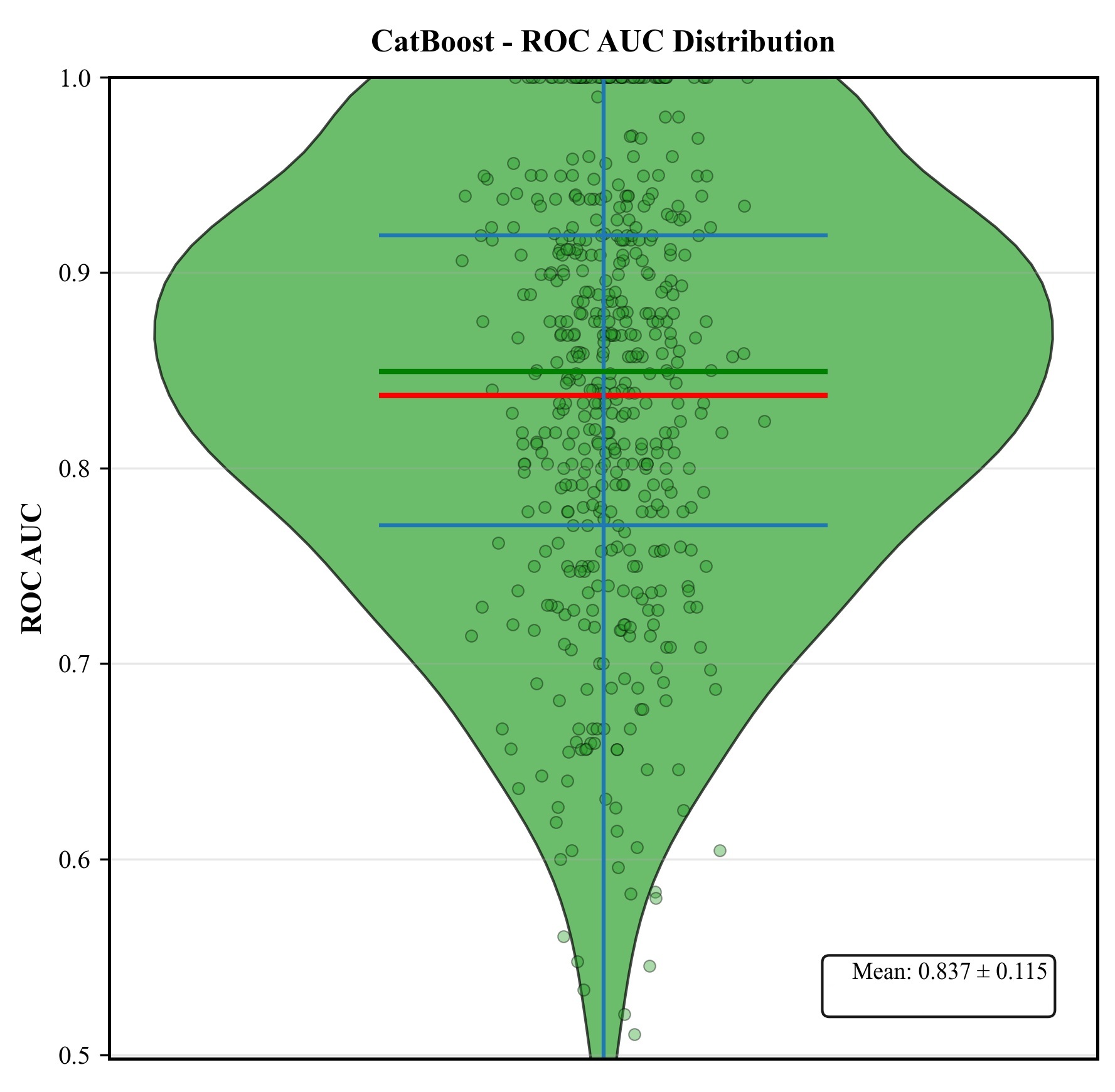

Supplement: Supplementary file 1 [file tomography-12-00029-s001.zip › Figure S4.jpg]

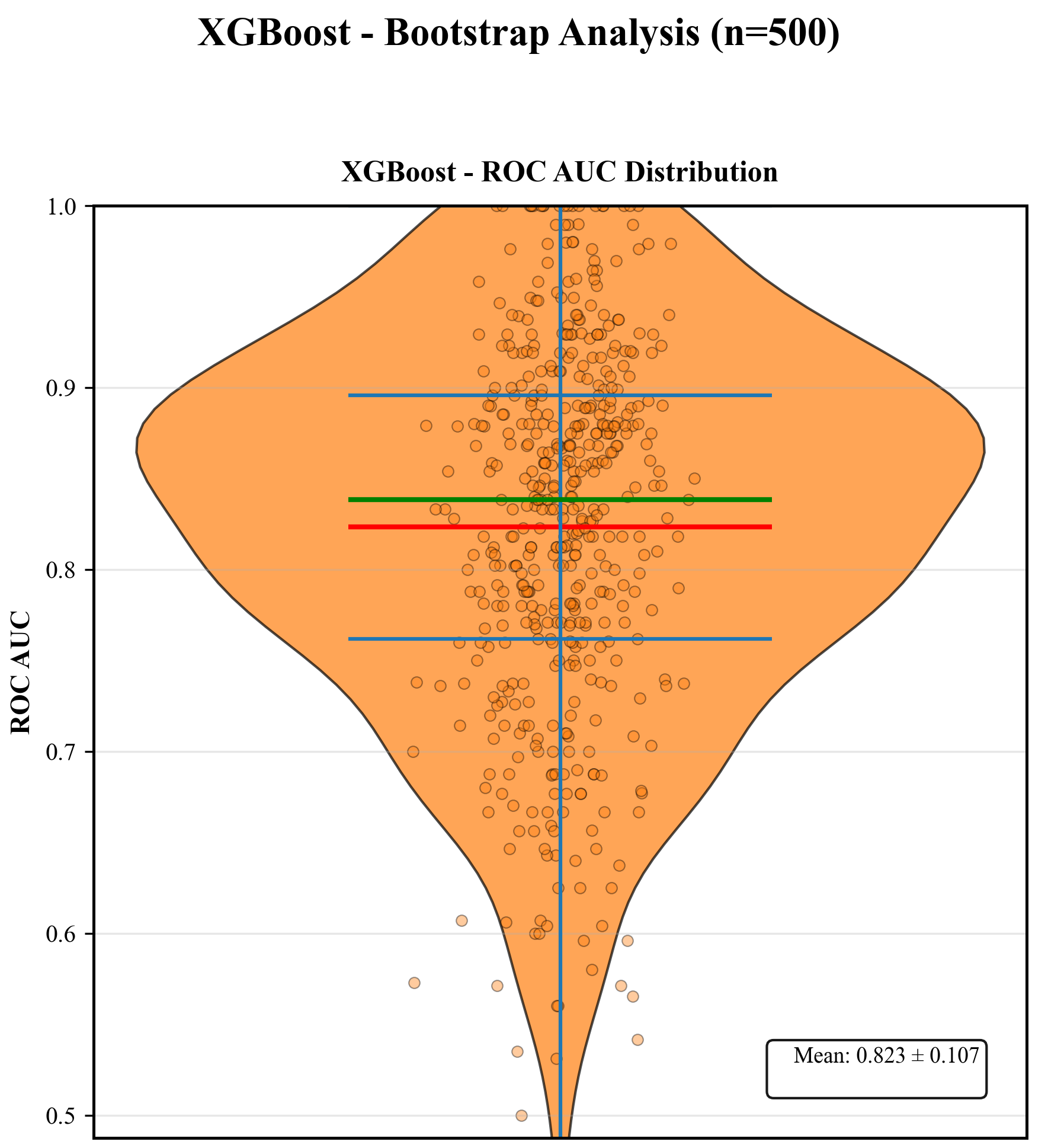

Supplement: Supplementary file 1 [file tomography-12-00029-s001.zip › Figure S5.png]

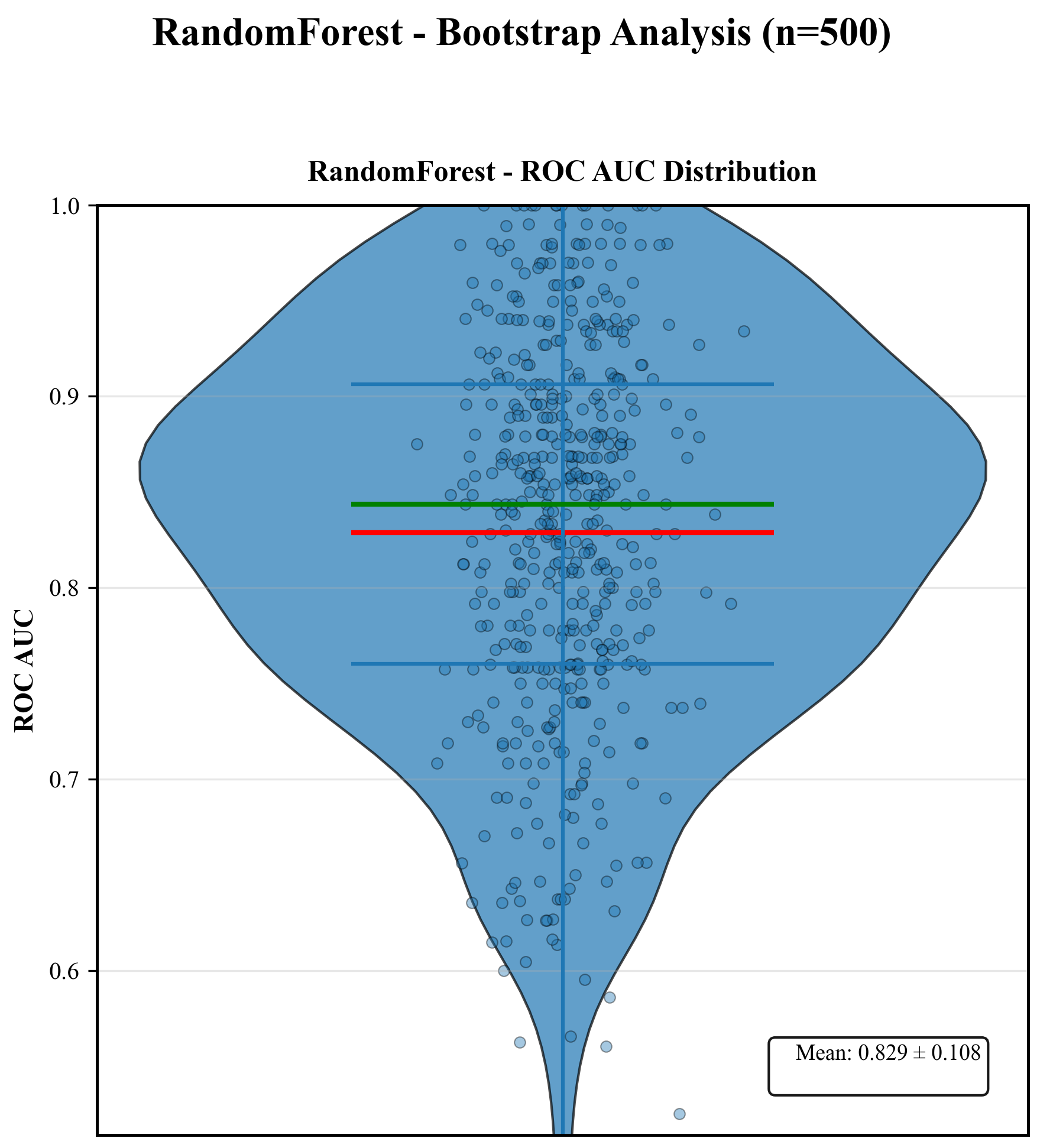

Supplement: Supplementary file 1 [file tomography-12-00029-s001.zip › Figure S6.png]

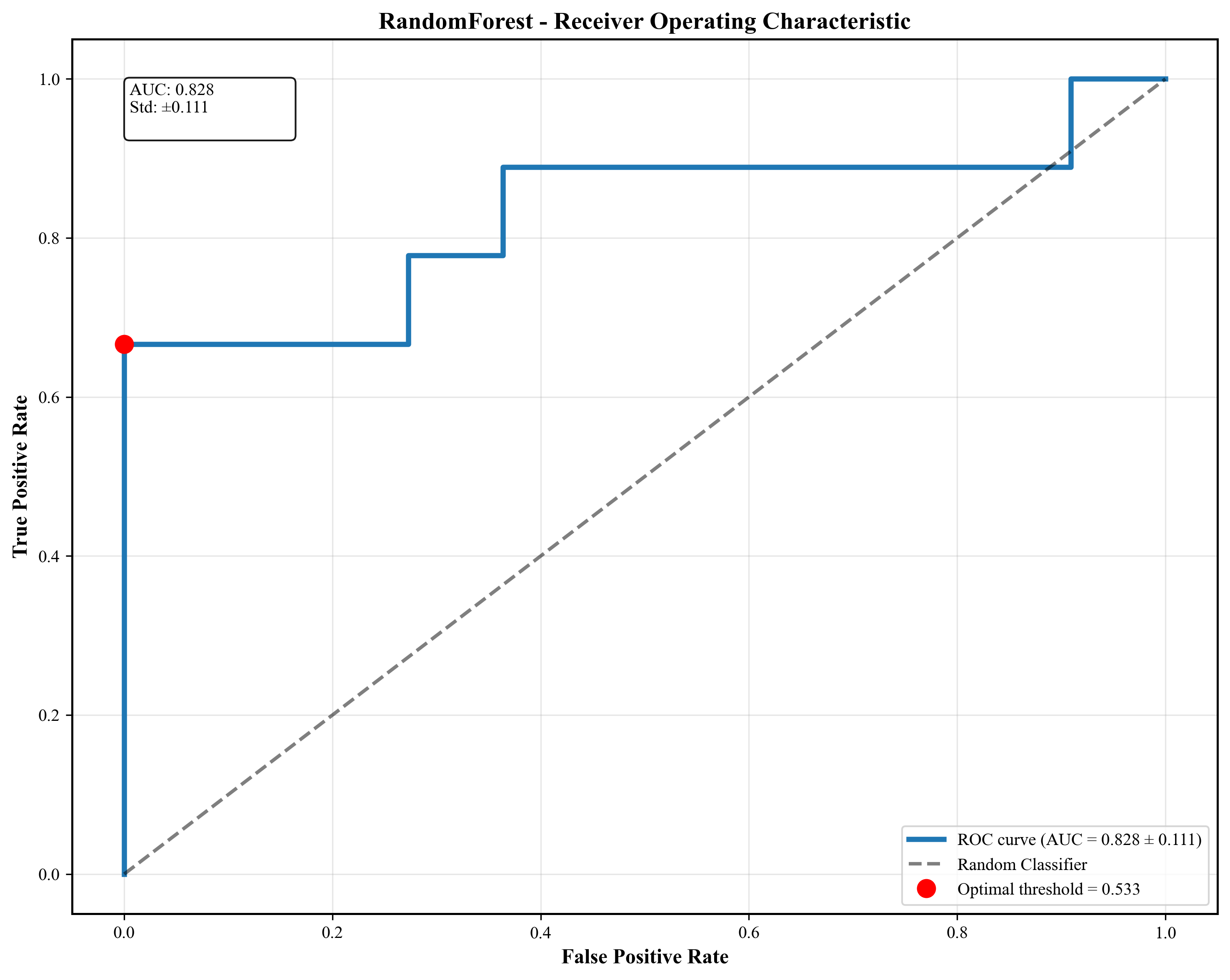

Supplement: Supplementary file 1 [file tomography-12-00029-s001.zip › Figure S7.png]

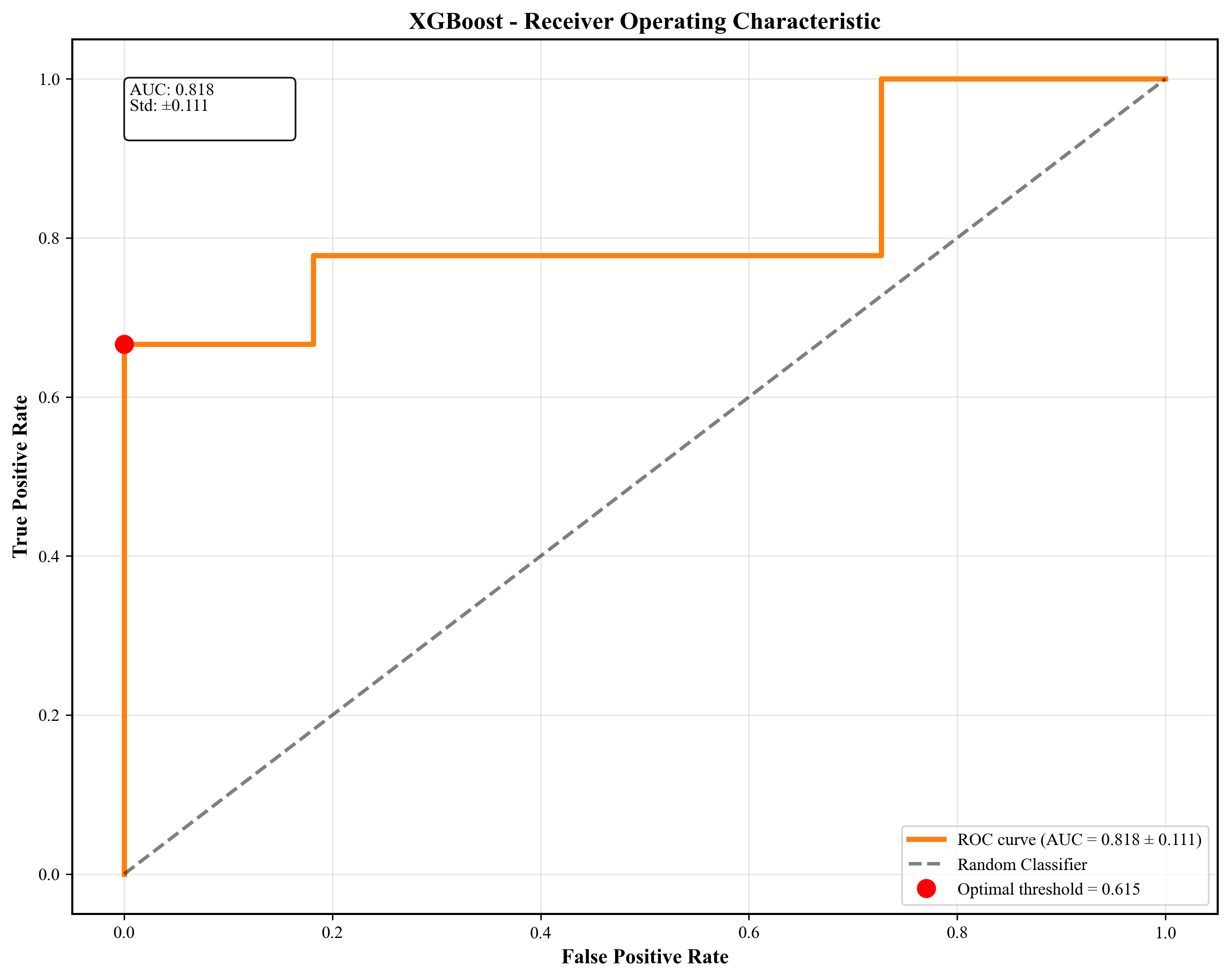

Supplement: Supplementary file 1 [file tomography-12-00029-s001.zip › Figure S8.png]

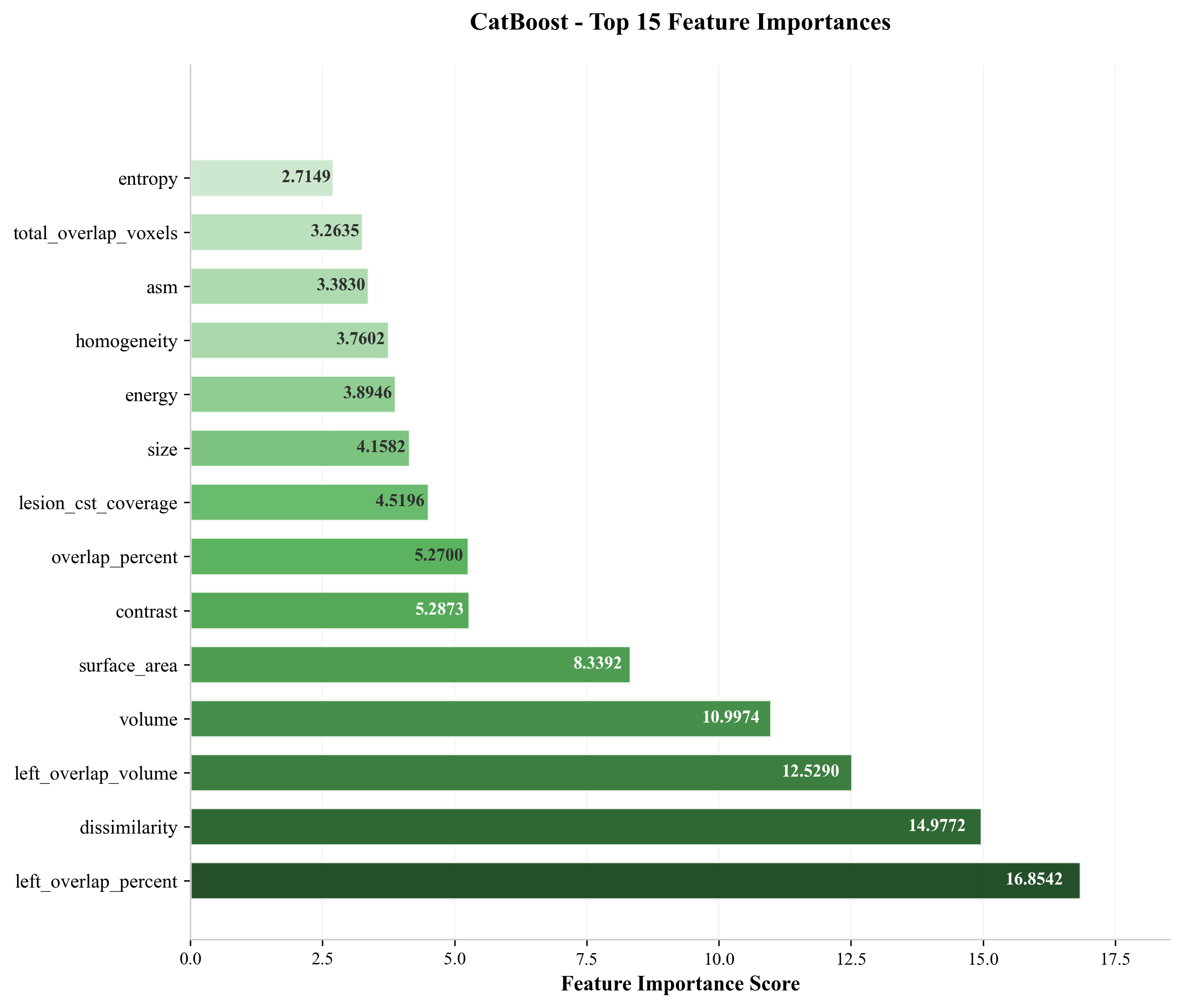

Supplement: Supplementary file 1 [file tomography-12-00029-s001.zip › Figure S9.png]
